# RBFOX2/GOLIM4 Splicing Axis Activates Vesicular Transport Pathway to Promote Nasopharyngeal Carcinogenesis

**DOI:** 10.1002/advs.202004852

**Published:** 2021-06-28

**Authors:** Chun‐Ling Luo, Xiao‐Chen Xu, Chu‐Jun Liu, Shuai He, Jie‐Rong Chen, Yan‐Chun Feng, Shu‐Qiang Liu, Wan Peng, Ya‐Qing Zhou, Yu‐Xiang Liu, Pan‐Pan Wei, Bo Li, Hai‐Qiang Mai, Xiao‐Jun Xia, Jin‐Xin Bei

**Affiliations:** ^1^ Sun Yat‐sen University Cancer Center State Key Laboratory of Oncology in South China Collaborative Innovation Center for Cancer Medicine Guangdong Key Laboratory of Nasopharyngeal Carcinoma Diagnosis and Therapy Guangzhou 510060 P. R. China; ^2^ Department of Biochemistry and Molecular Biology Zhongshan School of Medicine Sun Yat‐sen University Guangzhou 510080 P. R. China; ^3^ RNA Biomedical Institute Sun Yat‐sen Memorial Hospital Sun Yat‐sen University Guangzhou 510120 P. R. China; ^4^ Department of Experimental Research Sun Yat‐sen University Cancer Center Guangzhou 510060 P. R. China; ^5^ Department of Medical Oncology National Cancer Centre of Singapore Singapore 169610 Singapore

**Keywords:** alternative splicing, GOLIM4, nasopharyngeal carcinoma, RAB26, RBFOX2

## Abstract

20–30% of patients with nasopharyngeal carcinoma (NPC) develop distant metastasis or recurrence leading to poor survival, of which the underlying key molecular events have yet to be addressed. Here alternative splicing events in 85 NPC samples are profiled using transcriptome analysis and it is revealed that the long isoform of *GOLIM4* (‐L) with exon‐7 is highly expressed in NPC and associated with poor prognosis. Lines of evidence demonstrate the pro‐tumorigenic function of GOLIM4‐L in NPC cells. It is further revealed that RBFOX2 binds to a GGAA motif in exon‐7 and promotes its inclusion forming *GOLIM4‐L*. RBFOX2 knockdown suppresses the tumorigenesis of NPC cells, phenocopying GOLIM4‐L knockdown, which is significantly rescued by GOLIM4‐L overexpression. High expression of RBFOX2 is correlated with the exon‐7 inclusion of *GOLIM4* in NPC biopsies and associated with worse prognosis. It is observed that RBFOX2 and GOLIM4 can influence vesicle‐mediated transport through maintaining the organization of Golgi apparatus. Finally, it is revealed that RAB26 interacts with GOLIM4 and mediates its tumorigenic potentials in NPC cells. Taken together, the findings provide insights into how alternative splicing contributes to NPC development, by highlighting a functional link between GOLIM4‐L and its splicing regulator RBFOX2 activating vesicle‐mediated transport involving RAB26.

## Graphical abstract

Alternative splicing events are profiled in nasopharyngeal carcinoma (NPC) using transcriptome analysis, and a high expression of the long isoform of GOLIM4 containing exon‐7 (GOLIM4‐L) to be associated with poor survival of NPC is identified. A functional link between GOLIM4‐L and its splicing regulator RBFOX2 is also revealed, which activates vesicle‐mediated transport through maintaining Golgi complex integrity involving RAB26.

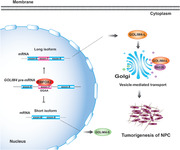

## 1 Introduction

Nasopharyngeal carcinoma (NPC) is a malignancy originated from the nasopharynx epithelium, with particularly high prevalence in Southern China, South‐eastern Asia, and Northern Africa.^[^ ^1^, ^2^ ^]^ The annual incidence rate is 25–30 per 100 000 in the endemic regions in Southern China.^[^ ^3^ ^]^ The 5‐year overall survival rate for patients with NPC has been improved recently, reaching 80%,^[^ ^4^ ^]^ largely due to the widespread implementation of intensity‐modulated radiotherapy and the addition of platinum‐based chemotherapy in patients with loco‐regionally advanced disease.^[^ ^2^ ^]^ Nonetheless, approximately 20–30% of patients develop distant metastasis or loco‐regional recurrence eventually leading to death.^[^ ^5^ ^]^ Therefore, it is important to characterize key molecular events responsible for the development of NPC, so as to identify effective targets for precise treatment against NPC.

Alternative pre‐mRNA splicing (AS) is a critical post‐transcriptional progress generating multiple mature mRNA transcripts that encode protein isoforms with disparate structure and function.^[^ ^6^ ^]^ Accumulating evidence has demonstrated that aberrant AS is a common process in multiple types of cancer,^[^ ^7^, ^8^ ^]^ thereby generating diverse gene variants involved in the proliferation and invasiveness of cancer cells.^[^ ^9^, ^10^, ^11^ ^]^ Generally, AS process is regulated strictly and precisely by regulatory proteins and intrinsic *cis*‐acting RNA elements, which determine an alternative exon to be included or excluded.^[^ ^12^ ^]^ Failure to recognize splice sites accurately due to either the mutations or dysfunctions of splicing regulators would lead to aberrant AS, which generates abnormal mRNA isoforms. These isoforms may exhibit different functions or even loss of function, and substantially contribute to cell abnormalities and malignancy.^[^ ^13^ ^]^ RNA binding proteins (RBPs) are important splicing regulators implicated in cancers, of which dysregulated expression results in dramatic changes of AS profiles in cells.^[^ ^11^, ^14^ ^]^ However, whether aberrant AS and the corresponding splicing regulators play functional roles in NPC development has not been addressed.

Here we profiled alternative splicing events in NPC tumor samples using transcriptome analysis, which revealed an alternative splicing variant of *GOLIM4* to be associated with the survival of patients with NPC. We further carried out functional characterization of GOLIM4 and identified RBFOX2 as an RNA binding protein mediating its alternative splicing. We also identified a functional RBFOX2/GOLIM4 axis that regulated their tumorigenic potentials through activating Golgi apparatus mediated vesicular transport involving RAB26. Our data provides evidence that alternative splicing plays an important role in the development of NPC.

## 2 Results

### 2.1 Landscape of AS Events in NPC

**Figure 1:**
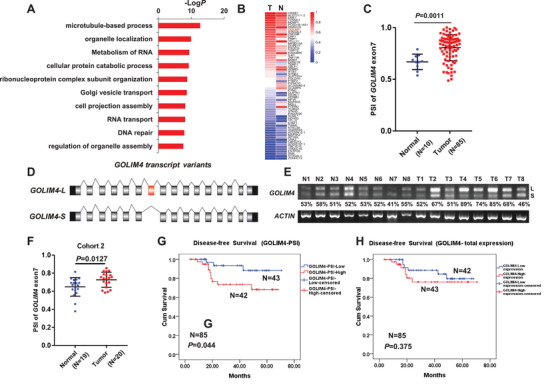
Identification of alternative splicing of *GOLIM4* related to NPC progression. A) Gene ontology analysis of aberrant alternative splicing events in NPC tissues (*n* = 85) and control samples (*n* = 10). B) Heatmap of percent spliced‐in index (PSI) for 63 splicing events in the above samples. C) High PSI of *GOLIM4* with the exon‐7 in the above NPC samples. D) Schematic diagram of *GOLIM4* splicing variants. Constitutive exons are shown as grey and black boxes, with black, grey, and red boxes for untranslated regions, coding regions, and the alternative exon‐7, respectively. E) RT‐PCR showed the transcription of the splicing variants of *GOLIM4* in NPC biopsies (T, *n* = 20) and control rhinitis tissues (N, *n* = 19). PSI value of each sample was shown at the bottom. *ACTIN* was used as control. F) qRT‐PCR showed the transcription levels of *GOLIM4* variants in samples described in (E). G,H) Kaplan–Meier survival curves of disease‐free survival for patients with NPC grouped by PSI level of *GOLIM4*‐*L* and the total mRNA expression level of *GOLIM4*. *p < 0.05, **p < 0.01, ***p < 0.001.

To profile the global AS events that might be linked to NPC progression, we first analyzed whole transcriptome data of 95 samples including NPC tumors (*n* = 85) and noncancerous control samples (*n* = 10). We observed large numbers of AS events involving 9757 genes in the NPC and the control samples. Survival analysis revealed that 1506 genes were significantly associated with the disease‐free survival (DFS) of patients with NPC. Gene ontology (GO) pathway analysis showed that most of these genes were involved in organelle localization, RNA metabolism and other RNA related pathways (**Figure** **1**A). Among them, 239 genes were found significantly dysregulated in the NPC samples compared with the control samples (Wilcox Test *p* < 0.05). To identify specific AS events that might contribute to NPC progression, we prioritized candidate genes based on the percent spliced‐in index (ΔPSI, indicating how efficiently sequences of interest are spliced in transcripts). Thus, we identified 63 genes with abnormal AS, of which dysregulation might be related to NPC progression (Figure 1B; Table S1, Supporting Information).

### 2.2 GOLIM4‐L Is Highly Expressed in NPC and Associated with Poor Prognosis of Patients with NPC

Subsequently, we further shortlisted the 63 genes with high PSI in the tumor samples, preferred locations of alternative exon in the coding region, appropriate length of the alternative exon (30–300 bp), and successful validation of AS events using reverse transcription‐PCR (RT‐PCR), which revealed *GOLIM4* as the gene of interest (Figure 1C). Human *GOLIM4* has 16 exons, and the AS event results in long (L) or short (S) isoforms with the inclusion or exclusion of the 84 bp exon‐7, respectively (Figure 1D). To determine the expression levels of *GOLIM4* variants in NPC samples, we performed RT‐PCR using a primer pair spanning the alternative exon‐7 (Figure S1A, Supporting Information). We observed that the ratio of *GOLIM4‐L* isoform was higher in the tumor samples compared with the control samples (Figure S1B, Supporting Information), consistent with the higher PSI observed in NPC tumors (Figure 1C), which were further validated in another independent collection of NPC (*n* = 20) and noncancerous (*n* = 19) samples (Figure 1E,F; Figure S1C, Supporting Information). Survival analysis revealed that higher PSI of *GOLIM4* meaning higher percentage of *GOLIM4‐L* was closely associated with poor survival in patients with NPC (Figure 1G; Figure S1D, Table S2, Supporting Information), while such associations were not observed for the total mRNA expression of *GOLIM4* (Figure 1H; Figure S1E, Table S2, Supporting Information). These observations suggest that *GOLIM4* splicing event might be a novel prognostic factor for patients with NPC and play an important role in NPC progression.

### 2.3 GOLIM4‐L Promotes Proliferation and Migration of NPC Cells In Vitro

**Figure 2:**
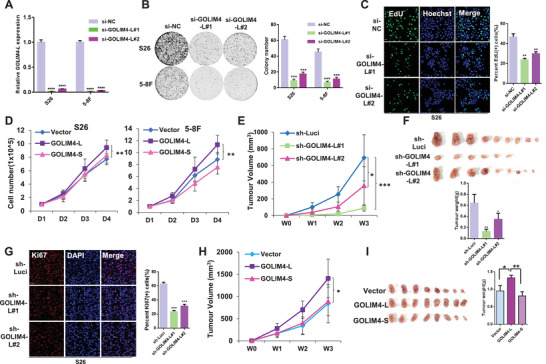
GOLIM4‐L promotes proliferation and tumorigenesis of NPC cells in vitro and in vivo. A) Human NPC cell lines S26 and 5–8F were transiently transfected with two siRNAs against *GOLIM4‐L* variant. *GOLIM4‐L* knockdown was assessed using qRT‐PCR. B) Colony formation assays were performed with cells described in (A). Three independent experiments were performed, and representative cells stained with crystal violet were shown (left). The number of focal adhesions was qualified, and results were shown as mean ± SD (Standard Deviation; right). C) Representative images of EdU staining assay for cells described in (A) (left). Data at the right are presented as mean ± SD. D) Cell growth curves were determined for S26 and 5–8F cells infected with lentivirus‐expressing GOLIM4‐L/S or empty vector control as indicated. E) Tumor growth of xenograft derived from S26 cells infected with sh‐GOLIM4‐L#1, sh‐GOLIM4‐L#2, and sh‐Luci lentivirus at different time courses. Tumor volumes were measured every week. F) Tumor size (upper) and weight (bottom) for the xenograft excised from (E). G) Fluorescent staining of Ki67 and DAPI in formalin‐fixed paraffin‐embedded tumor samples derived from (F). H) Tumor growth of xenograft derived from S26 cells with stable overexpression of GOLIM4‐L or GOLIM4‐S, or with control vector at different time courses. I) Tumor size (left) and weight (right) for the xenograft excised from (F). Scale bar, 100 µm. *p < 0.05, **p < 0.01, ***p < 0.001, ****p < 0.0001.

To investigate whether GOLIM4‐L contributes to the tumorigenesis of NPC, we specifically knocked down the expression of GOLIM4‐L in two NPC cell lines (S26 and 5–8F) using siRNAs targeting the exon‐7 of *GOLIM4*, which did not influence the *GOLIM4‐S* isoform (**Figure** **2**A; Figure S2A,B, Supporting Information). Strikingly, we observed significant inhibition in the growth and colony formation of the NPC cells with GOLIM4‐L knockdown compared with the control cells (Figure 2B; Figure S2C, Supporting Information). Moreover, confocal analysis showed that the known markers of cell proliferation, EdU(+) and Ki‐67(+), were significantly reduced in the GOLIM4‐L knockdown cells compared with the control cells (Figure 2C; Figure S2D, Supporting Information). These observations suggest that GOLIM4‐L knockdown inhibited the proliferation of NPC cells. To validate these findings, we also stably knocked down the expression of GOLIM4‐L in the two cell lines using shRNAs targeting the exon‐7 (Figure S3A, Supporting Information), which revealed consistent inhibitory effects of GOLIM4‐L knockdown on cell growth and colony formation in NPC cells (Figure S3B,C, Supporting Information). Moreover, wound healing and transwell assays showed that the migration ability of NPC cells was limited when GOLIM4‐L was stably knocked down (Figure S3D, Supporting Information). In addition, western blotting analysis showed that the expression of cell proliferation markers (CCND1 and c‐MYC) and migration related markers (N‐Cadherin and Vimentin) were decreased in the GOLIM4‐L knockdown cells compared with the control cells (Figure S3E, Supporting Information). These results strongly suggest that GOLIM4‐L might be essential for the proliferation and migration of NPC cells.

To further verify these findings, we generated NPC cell lines with stable overexpression of GOLIM4‐L or GOLIM4‐S (Figure S4A, Supporting Information). Cell growth and colony formation assays revealed that GOLIM4‐L overexpression significantly promoted the proliferation of NPC cells, while GOLIM4‐S overexpression had no such effects, as compared with the control groups (Figure 2D; Figure S4B, Supporting Information). Consistently, we observed that cells with EdU(+), the proliferation indicator, were significantly increased in the GOLIM4‐L overexpression group (Figure S4C, Supporting Information). Moreover, western blotting assay showed that CCND1 and c‐MYC were increased in the NPC cells overexpressing GOLIM4‐L compared with the GOLIM4‐S overexpression and the control groups (Figure S4E, Supporting Information). Furthermore, transwell assays revealed stronger migration ability of the NPC cells overexpressing GOLIM4‐L compared with the NPC cells with either GOLIM4‐S or empty vector (Figure S4D, Supporting Information). Consistently, we observed that the protein level of Vimentin was increased in the cells overexpressing GOLIM4‐L compared with that in the control and GOLIM4‐S overexpression groups (Figure S4E, Supporting Information). The promoting effects of GOLIM4‐L overexpression are opposite to that observed in NPC cells with GOLIM4‐L knockdown, which confirmed that GOLIM4‐L is essential to promote the cell proliferation and migration of NPC cells.

### 2.4 GOLIM4‐L Promotes Tumorigenesis of NPC Cells In Vivo

We next examined the role of GOLIM4‐L in tumorigenesis using in vivo xenograft models. S26 cells with stable knockdown of GOLIM4‐L using shRNAs targeting GOLIM4‐L (#1 or #2) or with control shRNA (sh‐Luci) were independently injected into nude mice. We observed significantly decreased tumor growth rate and tumor weight in the two GOLIM4‐L knockdown groups compared with the control group (Figure 2E,F). We also observed decreased proliferative cells as indicated by Ki67+ staining in the xenografts originated from the GOLIM4‐L knockdown cells compared with that from the control cells (Figure 2G). On the other hand, we independently inoculated S26 cells with stable overexpression of GOLIM4‐L or GOLIM4‐S, or empty control vector into nude mice. We observed higher tumor growth rate and tumor weight in the GOLIM4‐L overexpression group, while GOLIM4‐S overexpression had no obvious effects, as compared with the control group (Figure 2H,I). These in vivo data strongly confirmed the pro‐tumorigenic function of GOLIM4‐L in NPC cells, which is not present in the alternative GOLIM4‐S isoform.

### 2.5 RBFOX2 Promotes Exon Inclusion of GOLIM4 through Binding to the Alternative Exon

**Figure 3:**
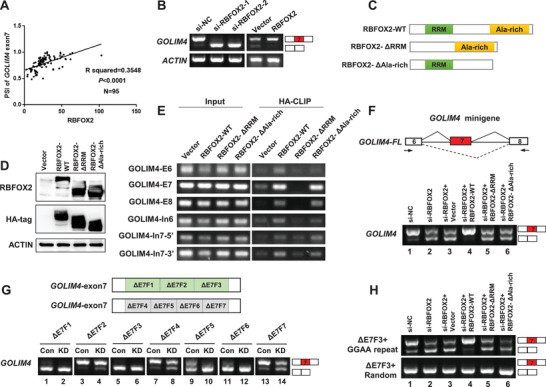
RBFOX2 promotes the inclusion of *GOLIM4* exon‐7 through binding to the alternative exon. A) Correlation between the PSI of *GOLIM4‐L* and the mRNA level of *RBFOX2* in NPC tissues (*n* = 85) and control samples (*n* = 10). Correlation coefficient *R* squared and *p* values were based on Pearson's correlation test. B) RT‐PCR examination of *GOLIM4* variants in S26 cells with RBFOX2 knockdown using siRNAs or overexpression with lentivirus. C) Schematic diagram of RBFOX2 domains and constructs of two RBFOX2 mutants, *Δ*RRM (deleting RRM) and *Δ*Ala‐rich (deleting Ala‐rich). Both the mutants and RBFOX2‐WT were HA tagged. D) Western blotting assays showed the expression of RBFOX2, HA‐tagged proteins, and ACTIN in 293T cells. Cells were transiently transfected with plasmids described in (C) and empty vector as control. E) Verification of direct binding between proteins as indicated on top and endogenous *GOLIM4* RNA fragments using CLIP assays. F) Schematic diagram of *GOLIM4* minigene constructs. RT‐PCR was performed to examine the splicing pattern of *GOLIM4* in 293T cells, which were co‐transfected with GOLIM4 minigene plasmids along with DNA constructs for siRNAs targeting RBFOX2 or control, as well as wildtype or mutant RBFOX2 as indicated on top. G) Schematic diagram of *GOLIM4* minigene with indicated deletions tiling exon‐7 were generated (upper). RT‐PCR examined the transcription of *GOLIM4* variants in 293 T cells (bottom), which were co‐transfected with si‐RBFOX2 (KD, as Knockdown) or si‐NC (Con, as Control) along with *GOLIM4* mutant plasmids. H) RT‐PCR showed in vivo splicing of *GOLIM4* mutant constructs in 293T cells, which were co‐transfected with GOLIM4 mutant plasmids indicated to the left along with DNA constructs for siRNAs targeting RBFOX2 or control, as well as wildtype or mutant RBFOX2 as indicated on top.

To identify any regulatory trans‐acting factor(s) that might regulate the alternative splicing of the exon‐7 of *GOLIM4*, forming either *GOLIM4‐L* or *GOLIM4‐S* isoform, we first analyzed the crosslinking and immunoprecipitation (CLIP)‐seq data obtained from POSTAR2 database.^[^ ^15^ ^]^ A total of 30 RBPs were predicted with potential bindings to pre‐mRNA of *GOLIM4*. To further prioritize the candidate RBPs for *GOLIM4*, we revisited the transcriptome data and found significant correlations between the PSI of *GOLIM4* and the expression of six RBPs, among which RBFOX2 showed the most significant (**Figure** **3**A; Figure S5A, Supporting Information). Next, we knocked down the expression of RBFOX2 in NPC cell lines using siRNAs (Figure S5B, Supporting Information), and observed the alternative splicing event for *GOLIM4*, of which the exon‐7 was almost completely skipped or excluded, resulting in *GOLIM4‐S* isoform, as compared with the control groups (Figure 3B; Figure S5D, Supporting Information). In contrast, the exogenous overexpression of RBFOX2 led to obvious increasement of *GOLIM4‐L* inclusive of the exon‐7 in NPC cells (Figure 3B; Figure S5C,D, Supporting Information). Moreover, when any of the two functional domains of the exogenous RBFOX2 was mutated (RBFOX2‐ΔRRM or ‐ΔAla‐rich, Figure 3C,D), the promoting effect of RBFOX2 on the exon inclusion for *GOLIM4* was impaired (Figure S5E, Supporting Information). These observations strongly suggest that RBFOX2 could regulate the exon‐7 inclusion of *GOLIM4*. In addition, such effect was not observed when any other RBPs (YBX3 or ELAVL1) was knocked down in NPC cells (Figure S5F,G, Supporting Information).

To further explore the regulatory mechanism of RBFOX2 on the alternative splicing of *GOLIM4*, we first screened for the binding site(s) of RBFOX2 in *GOLIM4* pre‐mRNA. We transiently overexpressed wild‐type (RBFOX2‐WT) or mutants at any of the two functional domains of RBFOX2 (RBFOX2‐ΔRRM or ‐ΔAla‐rich) in 293T cells. CLIP and RT‐PCR showed that RBFOX2‐WT and ‐ΔAla‐rich strongly bound to the exon‐7, but RBFOX2‐ΔRRM (which lacks the RNA binding domain) did not (Figure 3E). We subsequently introduced a minigene reporter plasmid containing the genomic DNA fragment from the exon‐6 to ‐8 of *GOLIM4* (GOLIM4‐minigene, Figure 3F, top) and analyzed alternative splicing of the exogenous exons in 293T cells with knockdown or rescue the expression of RBFOX2. We observed that the GOLIM4‐minigene with the exon‐7 inclusion was dominant in the wild‐type 293T cells, and RBFOX2 knockdown in 293T cells significantly decreased such inclusion resulting in its shorter isoform (Figure 3F, bottom, lanes 2 vs 1). Strikingly, introducing exogenous intact RBFOX2 in the cells obviously reverted the exon‐7 exclusion to forming the long isoform (Figure 3F, bottom, lanes 4 vs 3), nor did mutant RBFOX2 without functional domains (Figure 3F, bottom, lanes 5, 6 vs 4). This observation is consistent with the splicing pattern of the endogenous *GOLIM4* regulated by RBFOX2, further confirming the regulation of RBFOX2 on the *GOLIM4* exon‐7.

In order to investigate the exact binding motif of RBFOX2 on *GOLIM4* pre‐mRNA, we next introduced multiple minigene constructs with deleted fragments tiling the exon‐7 (Figure 3G). We observed a large degree or complete exclusion of the exon‐7 in 293T cells with the ΔE7F1, ΔE7F3, ΔE7F5, or ΔE7F6 mutants (Figure 3G, lanes 1, 5, 9, and 11). By contrast, deletions either in exon‐6, exon‐8, or intron‐7 had no effect on *GOLIM4* splicing (Figure S6A,B, Supporting Information). Moreover, overexpression of the intact/wild‐type RBFOX2 significantly increased the exon‐7 inclusion in the cells with mutant ΔE7F1 and ΔE7F5 compared with the RBFOX2 proteins without functional domains, while such upregulatory effect of RBFOX2 on the exon‐7 inclusion was completely abolished in the cells with mutant ΔE7F3 and ΔE7F6 (Figure S6C, Supporting Information). These observations strongly suggest that the common deleted GGAA sequence in the ΔE7F3 and ΔE7F6 of *GOLIM4* is the essential binding motif to RBFOX2 (Figure S6D, Supporting Information). To verify the functional relevance of GGAA to mediating splicing, we inserted two GGAA repeats into ΔE7F3 and observed significant increasement of exon‐7 inclusion in the construct with GGAA repeats, but not that with the random sequence (Figure 3H, lanes 1). Importantly, we also observed the effect of RBFOX2 knockdown on the splicing out of exon‐7 with GGAA repeats was rescued by introducing intact/wild‐type RBFOX2 (Figure 3H, lanes 2 and 4, respectively), but not the RBFOX2 mutants absent of functional domains (Figure 3H, lanes 5 and 6 vs 4). Taken together, these observations strongly suggest that GGAA motif in the *GOLIM4* exon‐7 is an essential binding site, whereby RBFOX2 executes the splicing of the exon‐7, resulting in *GOLIM4‐L* or *GOLIM4‐S* isoform.

### 2.6 RBFOX2 Acts as a Tumorigenesis Regulator in NPC

**Figure 4:**
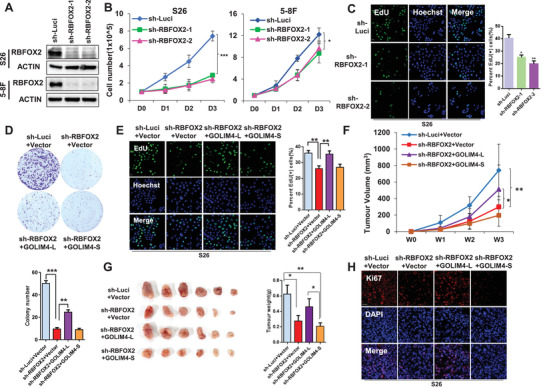
Overexpression of GOLIM4‐L rescues the inhibitory effects of RBFOX2 knockdown on NPC cells. A) Western blotting assay showed the expression of RBFOX2 and ACTIN in S26 and 5–8F cells transfected with sh‐RBFOX2‐1, sh‐RBFOX2‐2, or sh‐Luci lentivirus. B) Cell proliferation assays were performed using cells described in (A). C) Representative images of EdU staining assays (left) and the percentage of EdU positive cells (right) for cell lines described in (A). Data at the right are presented as mean ± SD. D) Colony formation assay was performed with S26 cells expressing shRBFOX2 or sh‐Luci (control) that infected with lentivirus expressing GOLIM4‐L, GOLIM4‐S or empty vectors. Quantification of colony numbers was shown at the bottom. Three independent experiments were performed, and error bars indicate SD of mean. E) Representative images of EdU staining assays (left) for cells described in (D) and corresponding statistics are shown at the right. F) Tumor growth of xenograft derived from S26 cells described in (D) at different time courses. G) Tumor size (left) and tumor weight (right) for xenograft excised from (F). H) Fluorescent staining of Ki67 and DAPI in formalin‐fixed paraffin‐embedded tumor samples derived from (G). Scale bar, 100 µm. *p < 0.05, **p < 0.01, ***p < 0.001.

In light of the pro‐tumorigenic function of GOLIM4‐L and the regulatory of RBFOX2 on the alternative splicing of *GOLIM4‐L*, we aimed to characterize the function of RBFOX2 on NPC cells. We stably knocked down the expression of RBFOX2 in NPC cells using shRNAs (sh‐RBFOX2‐1 and sh‐RBFOX2‐2, **Figure** **4**A) and observed significant reductions in cell growth and colony formation capacities of the NPC cells, compared with the control cells (Figure 4B; Figure S7A, Supporting Information). We also observed that the proliferation markers (EdU and Ki‐67) were strongly reduced in RBFOX2 knockdown cells (Figure 4C; Figure S7B, Supporting Information). Moreover, wound healing and transwell assays showed that RBFOX2 knockdown obviously inhibited the migration of NPC cells (Figure S7C,D, Supporting Information). These data suggest that RBFOX2 is essential for cell proliferation and migration in NPC cells, which is consistent with the effects of GOLIM4‐L.

### 2.7 Restoration of GOLIM4‐L Partially Rescues the Anti‐Tumorigenesis Effects of RBFOX2 Knockdown

To investigate the functional link between GOLIM4 and RBFOX2 contributing to tumorigenesis, we first generated stable NPC cell lines with RBFOX2 knockdown and meanwhile overexpression of GOLIM4‐L or GOLIM4‐S. We observed that RBFOX2 knockdown interrupted the formation of GOLIM4‐L in NPC cells (Figure S8A, Supporting Information) and led to the reduction of proliferating cells and numbers of survival colonies, which was partially restored by the overexpression of GOLIM4‐L but not GOLIM4‐S (Figure 4D,E). Similar rescue effect on migration ability was also observed in NPC cells with RBFOX2 knockdown and GOLIM4‐L overexpression (Figure S8B, Supporting Information).

To further validate the findings, in vivo mice model was applied. We observed that the xenografts with RBFOX2 knockdown exhibited obvious reduction in size and weight compared with control group (Figure 4F,G). Moreover, GOLIM4‐L overexpression in the xenografts with RBFOX2 knockdown significantly promoted tumorigenesis; however, the xenografts with GOLIM4‐S overexpression and RBFOX2 knockdown (Figure 4F,G) did not. Consistently, immunofluorescent analysis with Ki‐67 staining revealed that RBFOX2 knockdown significantly decreased proliferating cells in xenografts compared with the control group, and simultaneous overexpression of GOLIM4‐L, but not GOLIM4‐S, in the xenografts offset the inhibition caused by RBFOX2 knockdown (Figure 4H; Figure S8C, Supporting Information). These observations strongly suggest that RBFOX2 regulates the alternative splicing of *GOLIM4*, which in turn mediates its tumorigenesis functions in NPC cells.

### 2.8 High Expression of RBFOX2 Is Associated with Poor Prognosis of NPC

**Figure 5:**
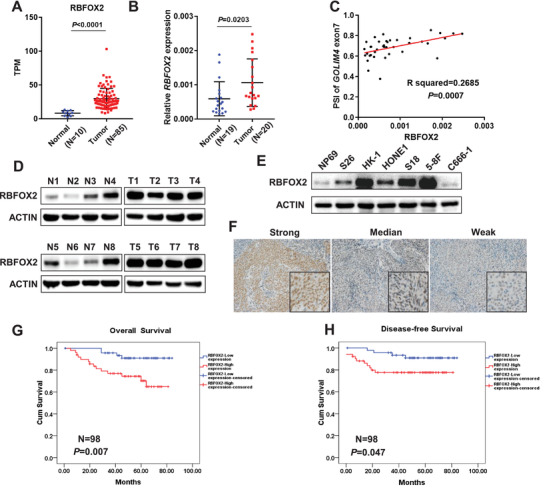
Expression of RBFOX2 in NPC and its association with prognosis of patients with NPC. A) Transcriptome analysis showed the mRNA expression of *RBFOX2* in NPC tissues (*n* = 85) and control samples (*n* = 10). B) qRT‐PCR showed the transcription level of *RBFOX2* (in relative to *ACTIN*) in another independent sample collection of NPC biopsies (*n* = 20) and control tissues (*n* = 19). C) Correlation between the PSI of *GOLIM4‐L* and the mRNA level *RBOFX2* in samples described in (B). D) Western blotting assays showed the protein expression of RBFOX2 in samples described in (B). ACTIN was used as control. E) Western blotting assays showed the protein levels of RBFOX2 and ACTIN in NPC cell lines and normal nasopharyngeal epithelium cells (NP69). F) Representative images of immunohistochemistry (IHC) assay for formalin‐fixed paraffin‐embedded NPC tissues (*n* = 98) with RBFOX2 antibody. Scale bar, 100 µm. G,H) Kaplan–Meier survival curves of overall and disease‐free survival in patients with NPC as described in (F) stratified by the protein level of RBFOX2. *p < 0.05, **p < 0.01, ***p < 0.001.

We next examined RBFOX2 expression in NPC biopsy samples. Transcriptome analysis revealed that RBFOX2 was significantly upregulated at mRNA level in NPC samples (*n* = 85) compared with noncancerous samples (*n* = 10, **Figure** **5**A), which was consistent with the observation in another sample collection as revealed by quantitative RT‐PCR (qRT‐PCR) (Figure 5B). Moreover, RBFOX2 expression was significantly correlated with the level of the exon‐7 inclusion in *GOLIM4* (Figure 5C). Western blotting assays showed that the protein expression of RBFOX2 was obviously augmented in NPC biopsies compared with noncancerous samples, as well as in NPC cell lines compared with transformed noncancerous nasopharyngeal cell line (NP69, Figure 5D,E). Immunohistochemistry (IHC) analysis revealed that RBFOX2 expression was mainly presented in tumor cells (Figure 5F). With that, Kaplan–Meier survival analysis revealed that high level of RBFOX2 protein was significantly associated with poorer overall survival and disease‐free survival for patients with NPC (Figure 5G,H). These observations are consistent with the pro‐tumorigenic role of RBFOX2 as revealed in the above in vitro and in vivo models, and further suggest that RBFOX2 is involved in the development of NPC.

### 2.9 RBFOX2 and GOLIM‐4 Might Regulate Vesicle‐Mediated Transport Pathway through Maintaining the Golgi Apparatus in NPC Cells

**Figure 6:**
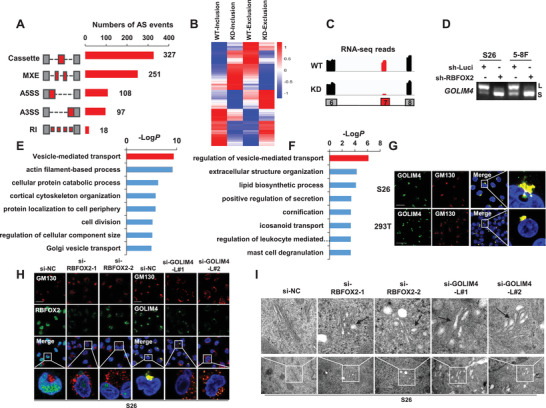
Pathways regulated by RBFOX2 and GOLIM4‐L in NPC cells. A) RBFOX2‐ affected alternative splicing events in S26 cells stably expressing RBFOX2‐shRNA and control shRNA (sh‐Luci). The events are classified into five categories: Cassette, mutually exclusive exon (MXE), alternative 5′‐splice site (A5SS), alternative 3′‐ splice site (A3SS) and retained intron (RI). B) Heatmap of alternative splicing events affected by RBFOX2 in above cells. The data were sorted by the mean value of WT (sh‐Luci) and KD (sh‐RBFXO2) groups. Red and blue indicated exon inclusion or exclusion, respectively. C) RNA sequencing reads mapping to *GOLIM4* in above cells. D) RT‐PCR examination of *GOLIM4* variants in S26 and 5–8F cells with RBFOX2 knockdown using shRNA. E) Significant gene ontology pathways affected by RBFOX2‐targeted splicing events. F) Significant biological processes involving genes with differential expression in S26 cells with GOLIM4‐L knockdown. G) Immunofluorescent staining showed the co‐localization of endogenous GOLIM4 (green) and GM130 (red) in S26 and 293T cells. Scale bar, 100 µm. H) Immunofluorescent staining of endogenous GM130 (red) together with RBFOX2 (green) or GOLIM4 (green) in S26 cells transfected with RBFOX2, GOLIM4‐L siRNAs or control siRNAs indicated on top. Scale bar, 100 µm. I) Electron microscopy showed the ultrastructure of Golgi in S26 cells transfected with siRNAs of RBFOX2, GOLIM4‐L, or control. Scale bar, 200 nm.

To explore the potential signaling pathways underlying the contributions of RBFOX2 and GOLIM4 in NPC, we performed transcriptome analyses on NPC cells with RBFOX2 or GOLIM4‐L knockdown. We observed a total of 801 AS events in response to RBFOX2 knockdown in S26 cells, with majority of cassette exons (327, Cassette), followed by mutually exclusive exons (251, MXE), alternative 5′‐ splice sites (108, A5SS), alternative 3′‐ splice sites (97, A3SS), and retained introns (18, RI, **Figure** **6**A). Noteworthy, most RBFOX2‐affected splicing events resulted in substantial changes in either inclusion or exclusion of alternative exons (Figure 6B). Among them, the exclusion of exon‐7 in *GOLIM4* was further validated (Figure 6C,D). GO analysis revealed that these RBFOX2‐affected targets were functionally associated with multiple pathways leading by vesicle‐mediated transport (Figure 6E). Additional GO analysis also revealed that vesicle‐mediated transport was among the top pathways involving genes regulated by GOLIM4‐L knockdown (Figure 6F).

Vesicular transport is a precisely organized cellular process involving the Golgi apparatus, endoplasmic reticulum, and lysosomes.^[^ ^16^ ^]^ As GOLIM4 is a Golgi integral membrane protein, we examined whether GOLIM4 regulates the Golgi apparatus in NPC cells. First, immunofluorescent staining and confocal microscopy revealed a colocalization of GOLIM4 (both GOLIM4‐L and GOLIM4‐S) and GM130 (known Golgi marker) in the Golgi apparatus in NPC cells and 293T cells (Figure 6G; Figure S9A,B, Supporting Information). Second, we observed a well‐assembled Golgi morphology reflected by GM130 staining in NPC cells and 293T cells, however, the Golgi apparatus was disassembled into fragments in the cytoplasm when RBFOX2 or GOLIM4‐L was knocked down in NPC cells (Figure 6H; Figure S9C,D, Supporting Information). Lastly, electron microscopy examination revealed a normal ultrastructure of Golgi apparatus, with a laterally connected, elongated, and flat cisternae morphology in the control cells; however, the inner lumen of the Golgi was swollen in the RBFOX2 or GOLIM4‐L knockdown cells (Figure 6I). These observations strongly suggest that RBFOX2 and GOLIM4 play important roles in regulating the assembly of Golgi apparatus.

In addition, to probe the potential mechanisms that Golgi fragmentation as a result of GOLIM4‐L knockdown modulated tumorigenesis in NPC cells, we performed immunoblotting array, revealing that the secretions of multiple chemokines related to tumorigenesis were significantly decreased in the GOLIM4‐L knockdown cells (Figure S10, Supporting Information).

### 2.10 GOLIM4‐L Interacts and Recruits RAB26 to Golgi and Mediate Vesicular Transport

**Figure 7:**
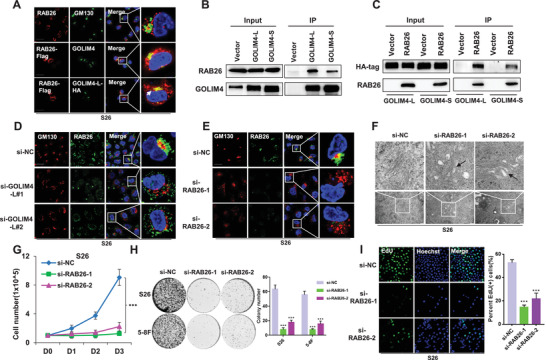
GOLIM4‐L binds and recruits RAB26 to mediate vesicle transportation. A) Immunofluorescent staining showed the localization of endogenous GM130 (green) and RAB26 (red), as well as colocalizations of exogenous RAB26 with Flag‐tag (red) and endogenous GOLIM4 (green) or exogenous GOLIM4‐L with HA‐tag (green). Scale bar, 100 µm. DNA constructs indicated on top were transiently introduced in 293T cells and whole cell lysis (input) or proteins immunoprecipitated (IP) with B) anti‐HA or C) anti‐FLAG antibody were immunoblotted with antibodies indicated at the left. Immunofluorescent staining of endogenous GM130 (red) and RAB26 (green) in S26 cells transfected with D) *GOLIM4‐L* siRNAs, or E) RAB26 siRNAs, or control siRNA. Scale bar, 100 µm. F) Electron microscopy showed the ultrastructure of Golgi in S26 cells described in (E). Scale bar, 200 nm. G) Cell proliferation assays were performed using S26 cells transfected with RAB26 siRNAs or control siRNA. H) Colony formation assays were performed with cells described in (E). Quantification of colony numbers was shown at the right. I) Representative images of EdU staining assays (left) for cells described in (E) and corresponding statistics are shown at the right. Scale bar, 100 µm. *p < 0.05, **p < 0.01, ***p < 0.001.

We validated three of the most downregulated genes in the vesicle‐mediated transport pathway (*C4BPB*, *RAB26*, and *CACNA1H*) derived from the GO analyses using qRT‐PCR, which revealed that RAB26 was significantly downregulated in the GOLIM4‐L knockdown cells (Figure S11A, Supporting Information). Consistently, western blotting assays showed that RAB26 was obviously decreased in NPC cells with RBFOX2 or GOLIM4‐L knockdown (Figure S11B, Supporting Information). RAB proteins are known as key regulators of Golgi apparatus organization and fragmentation.^[^ ^17^ ^]^ We observed co‐localization of RAB26 and GOLIM4‐L in the cytoplasm and partly in the Golgi apparatus (**Figure** **7**A). Moreover, co‐immunoprecipitation (co‐IP) assay strongly revealed that RAB26 could interact with GOLIM4‐L directly, of which interaction was obviously stronger than that with GOLIM4‐S (Figure 7B,C). Knockdown of GOLIM4‐L obviously decreased the Golgi localization of RAB26 in NPC cells (Figure 7D; Figure S11C, Supporting Information). Strikingly, immunofluorescence staining of GM130 and confocal microscopy revealed Golgi fragmentation in NPC cell lines with RAB26 knockdown (Figure 7E; Figure S11D,E, Supporting Information). Furthermore, electron microscopy demonstrated that RAB26 knockdown induced the swollen inner lumen of the Golgi in NPC cells, consistent with RBFOX2 or GOLIM4‐L knockdown (Figure 7F). These observations suggest that RAB26 interacts with GOLIM4 and jointly play an essential role in the maintenance of Golgi apparatus.

To explore how GOLIM4 regulates the transcriptional levels of RAB26 in NPC cells (Figure S11A, Supporting Information), we first shortlisted transcription factors (TFs) associated with RAB26 through public ChIP‐seq data (see the Experimental Section), revealing 197 candidate TFs. Moreover, in combination of transcriptional correlation analyses, only two TFs were found to be significantly correlated with RAB26 in two NPC cohorts, among which E4F1 was the stronger (Figure S12A, Supporting Information). Second, we observed a downregulatory or upregulated effect of E4F1 on the transcription and production of RAB26 in E4F1‐knockdown or E4F1‐overexpression NPC cells, respectively (Figure S12B–G, Supporting Information). Lastly, we observed that knockdown of GOLIM4‐L resulted in protein reduction but not mRNA expression of E4F1 in NPC cells (Figure S12H,I, Supporting Information). Taken together, these observations suggest that GOLIM4 might regulate RAB26 transcription through modulating the protein expression of E4F1 in NPC.

We further explored the tumorigenic potentials of RAB26 in NPC cells. Cell growth rate and colony formation ability were decreased in NPC cells with RAB26 knockdown compared with the control cells (Figure 7G,H; Figure S13A, Supporting Information), as were the cells with staining of EdU(+) and Ki‐67(+) (Figure 7I; Figure S13B, Supporting Information). Moreover, cell migration was inhibited in NPC cells with RAB26 knockdown (Figure S13C, Supporting Information). To confirm the functional links between RBFOX2, GOLIM4‐L, and RAB26 in NPC cells, we overexpressed RAB26 in cells with stable knockdown of RBFOX2 or GOLIM4‐L (Figure S13D, Supporting Information). We observed that the inhibitory effects on cell proliferation by RBFOX2 or GOLIM4‐L knockdown were significantly rescued with RAB26 overexpression, compared with the control group (Figure S13E,F, Supporting Information). In addition, we observed significant arguments of the protein levels of RAB26 and GOLIM4 in NPC samples compared with control samples, as well as in NPC cell lines compared with normal nasopharyngeal cell line (Figure S14A,B, Supporting Information). These observations strongly suggest that the pro‐tumorigenic functions of RBFOX2 and GOLIM4‐L might be mediated via RAB26, involving the regulation of Golgi apparatus and vesicle‐mediated transport.

## 3 Discussion

Here, for the first time to our knowledge, we profiled the global view of alternative splicing events in NPC through transcriptome analyses, which revealed multiple alternative splicing events to be dysregulated and associated with NPC progression. Among these events, *GOLIM4* was prioritized for further investigation. We performed multiple assays using in vitro and in vivo models and revealed that the long transcript isoform containing exon‐7 (*GOLIM4‐L*) was upregulated in NPC tumors and could promote tumorigenesis of NPC cells, whereas the short isoform *GOLIM4‐S* without the exon‐7 has no such effects. Consistently, a recent study demonstrated that knockdown of GOLIM4 inhibited the proliferation, apoptosis, and cell cycle progression of a human tongue and pharyngeal squamous carcinoma cell line,^[^ ^20^ ^]^ suggesting an oncogenic role of GOLIM4 in multiple cancers.

Our study further identified RBFOX2 as an essential modulator to regulate the alternative splicing of *GOLIM4*. RBFOX2 is a well‐characterized pre‐mRNA splicing regulator and has been implicated in organ development and cancer progression.^[^ ^21^ ^]^ Using in vivo CLIP and minigene reporter assays, we demonstrated that RBFOX2 could bind to the GGAA motif in *GOLIM4* exon‐7 and mediate its alternative splicing of *GOLIM4*. Consistently, it has been reported that RBFOX2 mediates alternative splicing in a position‐dependent manner, which triggers exon skipping or inclusion if their binding was at the upstream or downstream of an alternative exon, respectively.^[^ ^22^ ^]^ We note that GGAA motif is presented in 35.2% (115/327) genes regulated by RBFOX2 (data not shown), suggesting that GGAA sequence is an important motif of broad significance for RBFOX2 to bind and regulate alternative splicing. For the remaining genes, we suppose that other mechanisms may be involved in the AS regulation of RBFOX2, given the complexity of splicing regulation.

Moreover, we observed that RBFOX2 was highly expressed in NPC tumors and promoted the tumorigenesis of NPC cells, suggesting its important role in the development of NPC. Supportively, a previous finding showed that RBFOX2 could promote cellular invasion through regulating alternative splicing driven by epithelial–mesenchymal transition.^[^ ^23^ ^]^ Furthermore, we note that RBFOX2 and the long GOLIM4 variant share similar pro‐tumorigenic functions on NPC cells and the tumor suppressive function of RBFOX2 knockdown could be partially rescued by overexpression of GOLIM4‐L. In addition, we observed that high level of GOLIM4‐L or RBFOX2 in NPC biopsies had a significantly inferior survival outcome. These findings confirm the functional link between the two molecules and their interactive roles in the development of NPC.

Our transcriptome analysis revealed that various genes affected by either RBFOX2 or GOLIM4‐L were functionally associated with multiple pathways leading by vesicle‐mediated transport. Vesicular transport is a precisely orchestrated cellular process that is responsible for exchanging proteins and lipids among the plasma membrane and the organelles including the Golgi apparatus, endoplasmic reticulum, and lysosomes.^[^ ^16^ ^]^ Consistent with the functions of Golgi apparatus in vesicular transport, GM130 staining results demonstrated that both GOLIM4‐L and RBFOX2 deficiency induced severe fragmentation of Golgi apparatus in NPC cells. Further electron microscopy results emphasized the important role of RBFOX2 and GOLIM4‐L in the stable organization of Golgi apparatus, suggesting their essential roles in mediating vesicular transport in NPC. In addition, immunoblotting assay of human chemokines revealed that GOLIM4‐L knockdown leading to Golgi fragmentation reduced secretion of several cancer‐promoting chemokines, consistent with a previous observation that Golgi fragmentation and reduced secretion of cancer growth‐promoting factors inhibit the growth and metastasis of pancreatic cancer.^[^ ^24^ ^]^ These results strongly suggest that GOLIM4‐L may hold its tumorigenic potential through maintaining the integrity of Golgi complex that may influence the secretion of tumor‐promoting factors including chemokines.

The alteration of vesicular transport pathway has been linked to tumor development and progression.^[^ ^25^ ^]^ We observed that the expression of RAB26 was significantly downregulated by knockdown of RBFOX2 or GOLIM4‐L. RAB26 is one of the Rab family proteins (Rabs) that are master regulators of vesicular transport, maintaining Golgi architecture and membrane trafficking in eukaryotic cells.^[^ ^26^ ^]^ Consistently, we observed the colocalization of RAB26 and GOLIM4 in the Golgi apparatus in NPC cells, as well as a direct binding between RAB26 and GOLIM4‐L revealed by co‐IP assay. Since GOLIM4 is a type II Golgi‐resident protein without evidence being a TF to regulate gene transcription, we explored candidate TFs and revealed that GOLIM4‐L might regulate the transcription level of RAB26 through a transcription factor E4F1. Moreover, RAB26 knockdown led to Golgi fragmentation in NPC cells, as did the RBFOX2 or GOLIM4‐L deficiency. Furthermore, we observed that RAB26 was required for cell proliferation and migration of NPC cells, which is consistent with a previous finding in non‐small‐cell lung carcinoma.^[^ ^27^ ^]^ RAB26 overexpression partially rescued the inhibitory effects on cell proliferation by RBFOX2 or GOLIM4‐L knockdown. With these observations, it is plausible that RBFOX2 and GOLIM4‐L promote the development of NPC through a perturbation of vesicular transport pathway, involving binding and recruitment of RAB26 to Golgi.

**Figure 8:**
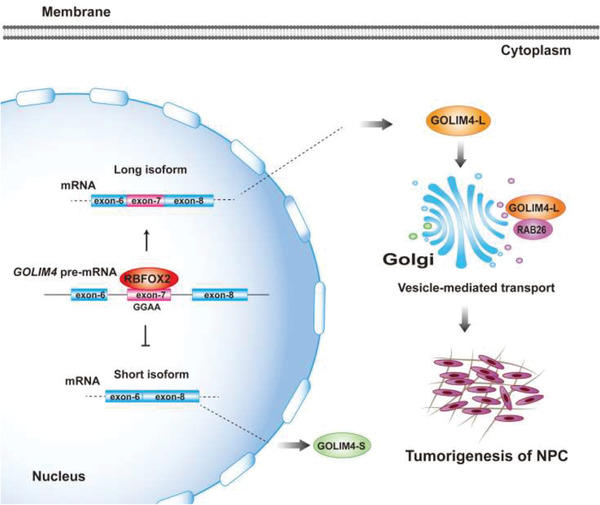
Schematic diagram showing a proposed model for the interactions among RBFOX2, GOLIM4, and RAB26 in NPC progression. RBFOX2 mediates the alternative splicing of the exon‐7 of *GOLIM4* through binding to GGAA sequence in the exon, which generates the long isoform of *GOLIM4‐L*. GOLIM4‐L promotes tumorigenesis of NPC, likely through vesicle‐mediated transport pathway involving recruitment of RAB26.

In summary, these data suggest a functional link among RBFOX2, GOLIM4, and RAB26, where RBFOX2 recognizes GGAA motif to generate a long isoform of *GOLIM4‐L* and jointly regulate the tumorigenesis of NPC cells through vesicle transportation, involving the recruitment of RAB26 to Golgi with a stronger binding to GOLIM4‐L (**Figure** **8**). Our findings provide insights into understanding the mechanisms in the development of NPC, where the dysregulation of alternative splicing as a critical posttranscriptional regulation of gene expression plays an important role; and our findings also provide insights into the development of clinical biomarkers and therapeutic approaches focusing on GOLIM4, RBFOX2, and vesicular transport pathway involving RAB26, of which clinical values await further validations in more patient cohorts. In addition, we acknowledged that the link among the three genes may describe a branch of the complicated mechanisms underlying Golgi fragmentation and tumorigenesis of NPC cells, since we did observe that RBFOX2 and GOLIM4 regulate multiple genes in addition to GOLIM4 and RAB26, respectively. Consistently, GOLIM4‐L and RAB26 could not fully rescue the cellular phenotypes caused by RBFOX2 or GOLIM4‐L knockdown, respectively. Given these and previous reports that aberrant alternative splicing events are commonly observed in cancer cells and linked to cancer development,^[^ ^8^, ^10^ ^]^ alternative splicing of other candidate genes in our study might also play important roles in NPC, which await further investigations.

## 4 Experimental Section

### Patient Recruitment and Samples Preparation

For whole transcriptome sequencing (WTS), patients with nasopharyngeal carcinoma (NPC, *n* = 85) or inflammation of nasopharynx (rhinitis, *n* = 10) were recruited at the Sun Yat‐sen University Cancer Center (SYSUCC), Guangzhou, China. For mRNA expression analysis, biopsies derived from patient with NPC (*n* = 20) and inflammation of nasopharynx (rhinitis, *n* = 19) were obtained independently at the SYSUCC. Biopsies were collected before any treatment and placed in RNAlater (Thermo Fisher Scientific, Waltham, MA, USA) and frozen at −80 °C. For immunohistochemistry, formalin‐fixed paraffin‐embedded (FFPE) biopsy specimens derived from patient with NPC (*n* = 98) were obtained at the SYSUCC. Patients with NPC were histopathologically diagnosed by at least two pathologists according to the WHO classification. Patients with rhinitis were outpatients with inflammatory diseases in the nasopharyngeal airway without NPC. Written informed consent was obtained from all participants, and the study was approved by the Institutional Review Boards at SYSUCC.

### Whole Genome Transcriptome Analysis

Total RNA samples from NPC tumors, noncancerous control tissues and cell lines were extracted and subsequently subjected for WTS as described previously.^[^ ^28^ ^]^ Briefly, total RNA was extracted from tumors with the RNeasy Mini Kit (Qiagen, Duesseldorf, Germany) and subjected to the depletion of ribosomal RNAs by using Ribo‐Zero Magnetic kit (Illumina, San Diego, California, USA), followed by library construction with TruSeq RNA Library Prep Kit (Illumina, San Diego, California, USA) according to the manufacturer's protocols. Subsequently, the library was processed with high‐throughput sequencing for pair‐end of 150 bp, using Hiseq X sequencer (Illumina, San Diego, California, USA). Another WTS dataset of NPC (*n* = 113) was retrieved from the public GEO database (Accession number: GSE102349, https://www.ncbi.nlm.nih.gov/geo/query/acc.cgi?acc=GSE102349).

High‐quality pair‐end reads were mapped using Bowtie 2.^[^ ^29^ ^]^ After filtering out ribosomal RNAs, reads counts of each gene were then quantitated using HTseq.^[^ ^30^ ^]^ The expression level of a gene was normalized using transcripts per million (TPM). Genes with low expression levels (mean of TPM <2) in all the data sets were removed from the downstream analysis. Reads mapping and data analysis for differentially regulated exons were carried out as previously described.^[^ ^11^ ^]^ PSI of GOLIM4 exon‐7 was calculated based on the number of reads supporting inclusion or exclusion events. Expression of RBFOX2 was calculated by counting the number of reads mapping to RBFOX2 gene. To prioritize the AS events of interest, the following exclusion criteria were applied: 1) without sufficient sequencing reads mapping to a candidate gene to calculate PSI in 2/3 of total samples (NA was assigned), 2) alternative splicing fragment of multiple 3 nt affecting less than five amino acids (AAs), 3) no significant association with disease‐free survival, 4) no significant difference of PSI between NPC and control samples, 5) NA of PSI in more than four control samples, and 6) change in PSI (ΔPSI) no more than 0.1).

### Cell Culture and Reagents

Human NPC cell lines (S26 and 5–8F) were kindly gifted by Professor Chaonan Qian at SYSUCC and human embryonic kidney 293T cells were purchased from Cell Bank of Type Culture Collection of Chinese Academy of Sciences, Shanghai Institute of Cell Biology, Chinese Academy of Sciences. All cell lines were cultured in Dulbecco's Modified Eagle Medium (DMEM, Gibco, NY, USA) supplemented with 10% fetal bovine serum (FBS, Gibco, NY, USA) and cell cultures were maintained in a 5% CO_2_ humidified incubator at 37 °C. No evidence of mycoplasma contamination was observed using mycoplasma detection kit (Vazyme, Nanjing, China). Primary antibodies were commercially available, including those for GOLIM4 (PAB28477, Abnova, Taipei, China), *β*‐actin (A1978, Sigma‐Aldrich, St. Louis, USA) and RBFOX2 (HPA006240, Sigma‐Aldrich, St. Louis, USA, ab57154, Abcam, Cambridge, USA), Tubulin, CCND1, C‐MYC, N‐Cadherin, and Vimentin (2144, 2922, 9402, 14215, 5741, Cell Signaling Technology, Danvers, USA), RAB26 (ab187151, ab198202, Abcam, Cambridge, USA), E4F1 (H00001877‐M03, Abnova, Taipei, China) and HA tag (ab9100, Abcam, Cambridge, USA, H6908, Sigma‐Aldrich, St. Louis, USA).

### Immunohistochemistry and Evaluation

IHC was performed to evaluate the protein expression of a candidate gene with a specific antibody against the protein as reported previously.^[^ ^11^ ^]^ In brief, paraffin sections were deparaffinized with xylene and rehydrated, followed by antigen retrieval. The sections were then treated with 3% hydrogen peroxide to quench the endogenous peroxidase activity, followed by incubation with a primary antibody overnight at 4 °C. After incubation with secondary antibodies for 1 h at room temperature, chromogenic immunolocalization was conducted using 0.05% 3,30‐diaminobenzidine (Dako, Glostrup, Denmark). All sections were counterstained with hematoxylin. IHC evaluation was performed independently by three pathologists at SYSUCC. An IHC staining score was calculated as the product of staining intensity multiplied by the percentage of stained cells (Table S3, Supporting Information). The staining intensity was evaluated on a scale of 0 to 3: 0 for no, weak, intermediate, and strong staining, respectively.

### siRNAs, Plasmids, and Lentivirus

The small interfering RNA (siRNA) oligos against GOLIM4‐L, RBFOX2, RAB26, and E4F1 were commercially synthesized by Gene Pharma (Shanghai, China), and transfected with Lipofectamine RNAiMAX (Invitrogen, Carlsbad, CA, USA). Plasmids containing HA‐tagged wild‐type RBFOX2 (RBFOX2‐WT) and RBFOX2 domain deletion mutants (RBFOX2‐ΔRRM or ‐ΔAla‐rich) were constructed using pcDNA3.1‐HA vector. GOLIM4 minigenes were constructed by amplifying genomic sequences spanning exons 6–8 of GOLIM4 gene, which were then cloned into pcDNA3.1 vector, separately. Deletion or insertion of mutant derivatives was prepared based on the minigene plasmids. Flag‐RAB26 was constructed with pCMV‐Tag2B vector. HA‐GOLIM4‐L/S were generated with pcDNA3.1 vector. DNA fragments for shRNAs targeting RBFOX2 (sh‐RBFOX2) and GOLIM4‐L (sh‐GOLIM4‐L) and full length cDNAs for GOLIM4‐L/S isoforms, RAB26 and E4F1 were independently cloned into PLKO.1‐puro and pCDH‐puro lentiviral vectors, respectively, which was packaged and transduced into 293T cells to generate respective lentivirus. S26 and 5–8F cells were infected with the lentivirus and selected against puromycin.

### Quantitative PCR and In Vivo Crosslinking and Immunoprecipitation

Total RNA was extracted from NPC tissue, noncancerous tissue, or cell lines, and was reverse transcribed using oligo (dT) primers and M‐MLV Reverse Transcriptase (Promega, Madison, WI, USA) according to the manufacturer's instructions. Real‐time quantitative PCR (qRT‐PCR) was performed to determine the transcription level of a gene using SYBR Premix Ex Taq kit (TaKaRa, Tokyo, Japan), and its corresponding primer pair (Table S4, Supporting Information). In vivo CLIP was carried out to detect RNA(s) binding to RBFOX2 or its mutants as previously described.^[^ ^11^ ^]^ In brief, 293T cells were transfected with HA‐tagged RBFOX2, mutant plasmids, or empty vector controls. The cells were exposed to ultraviolet light for crosslinking, followed by immunoprecipitation using Magna RIP kit (Merck Millipore, Billerica, MA, USA). RNA enrichment was measured by RT‐PCR with specific primers (Table S4, Supporting Information).

### Co‐IP and Western Blotting

Cells were lysed in cell lysis buffer (Cell Signaling Technology, Danvers, USA) with 1× protease inhibitor cocktail (Beyotime, Shanghai, China) for 30 min. After centrifugation, lysate protein concentration was quantified and then the lysate was incubated with indicate antibodies at 4 °C for 4 h. Subsequently, the pre‐cleared protein A/G beads (Sigma‐Aldrich, St. Louis, USA) were added to capture the antibody–protein complexes with overnight rotation at 4 °C. Next day, beads were washed with lysis buffer for five times, followed by protein elution in 1× sodium dodecyl sulfate sample buffer and boiling for 10 min. Then, samples were analyzed by western blotting. Total proteins in the supernatant fraction were loaded and separated by sodium dodecyl sulfate–polyacrylamide gel electrophoresis (SDS‐PAGE), followed by transfer to polyvinylidene difluoride (PVDF) membrane (Merck Millipore, Billerica, MA, USA). Blocking was done by immersing the membrane with 5% bovine serum albumin (BSA) in tris‐buffered saline and Tween 20 (TBST) buffer for 1 h at room temperature. The membrane was then immunoblotted with a primary antibody overnight at 4 °C, followed by incubation with a secondary monoclonal antibody of rabbit or mouse origin. The visualization of proteins was achieved by Fdbio‐Dura ECL kit (Fdbio science, Hangzhou, China) and Bio‐Rad ChemiDoc Touch (Hercules, CA, USA).

### Immunofluorescence Staining

Cells were fixed in 4% paraformaldehyde for 10 min at room temperature and rinsed with phosphate‐buffered saline (PBS) buffer for three times. Cells were then permeabilized using 0.1% Triton X‐100 in PBS for 10 min and washed three times with PBS. After blocking with 5% goat serum and Triton X‐100 for 1 h at room temperature, cells were incubated with primary antibodies as indicated overnight at 4 °C. After three washes with PBS, cells were incubated with fluorescent secondary antibodies in the dark for 1 h at room temperature, followed by three washes with PBS. Finally, cells were mounted with antifade mounting medium and 4′,6‐diamidino‐2‐phenylindole (DAPI) for 10 min. Immunofluorescence analysis was conducted on a confocal laser scanning microscope (Carl Zeiss, Microscope 880, Jena, Germany).

### Cell Proliferation and Colony Formation Assays

Cell proliferation was measured as the increasement of cell numbers at serial timepoints. Briefly, cells were plated in triplicate manner (1 × 10^5^ cells for per well) in a 12‐wells plate and cultured for 24, 48, and 72 h. At each timepoint, cell number and viability were determined using Trypan Blue exclusion and a hemocytometer under a light microscope. For colony formation assay, cells were seeded in triplicate (2000 cells per well for 6‐well plates) and were grown for 8–10 days with normal medium. Cells were fixed in 4% paraformaldehyde for 10 min at room temperature and stained with crystal violet solution, followed by cell enumeration using a light microscope. The colony numbers were determined by counting the number of colonies from six equal areas randomly each sample.

### Cell Migration Assays

For wound healing assay, 1 × 10^5^ cells were plated in triplicates in each well with a culture‐insert, which was removed after cultivation for 12 h, creating a defined cell‐free gap. Images were captured at 0, 24, and 48 h after removal of the insert, using an inverted microscope (IX73, Olympus, Tokyo, Japan) and the migration ability of the cells was calculated as the area measured in pixels between the edges of the scratching at the indicated time point in relative to that of the starting time point. For transwell assay, 5 × 10^4^ cells with serum‐free medium were seeded in triplicates into transwell chambers (8 µm pores, Corning, NY, USA) without Matrigel, and the chamber was put in 24‐well plates, where 500 µL DMEM containing 10% FBS was added to the lower chambers. After 24 h, cells on the membrane were fixed in 4% paraformaldehyde for 10 min and stained with crystal violet solution and subsequently enumerated under a light microscope.

### Electron Microscopy

S26 cells were transfected with siRNAs of RBFOX2, GOLIM4‐L, RAB26, or control for 48 h. Then cells were digested and centrifuged at lower speed (3000 rpm min^−1^) for 30 min to collect the precipitate. The samples were fixed with 2.5% glutaraldehyde in phosphate buffer at 4 °C overnight and then subjected for electron microscopy (JEM‐1400flash) at Sun Yat‐Sen University for subsequent processing and analysis.

### Human Chemokine Array Analysis

Human chemokine antibody arrays were purchased from RayBiotech (Cat. No. AAH‐CHE‐1, Norcross, GA, USA) and used according to the manufacturer's protocols. Briefly, 2 × 10^6^ S26 cells infected with lentivirus expressing GOLIM4‐L shRNA or control shRNA (sh‐Luci) were plated in 100 mm cell culture dishes. After 24 h, cells were washed with 1× PBS and added with 10 mL reduced‐serum OPTI‐MEM medium in each dish for 72 h. Then cell culture supernatants were collected and concentrated using centrifugal concentrator (Thermo Savant ISS110P1‐230). The array membranes were blocked with blocking buffer at room temperature for 0.5 h and then incubated with 1 mL concentrated cell culture supernatants at 4 °C overnight. After five washes with washing buffer, the membranes were incubated with primary biotin‐conjugated antibody cocktail at 4 °C overnight. Next day, the membranes were washed and incubated with 2 mL 1000‐fold diluted HRP‐conjugated streptavidin at room temperature for 2 h. Immunoblotting signals were detected using detection buffer and Bio‐Rad ChemiDoc Touch (Hercules, CA, USA). The signal intensities were quantified using Image J. Positive controls on the array membranes were used to normalize the results across different membranes.

### In Vivo Xenograft Assay

Male BALB/c nude mice of four‐week‐old were purchased from Beijing Vital River Laboratory Animal Technology (Beijing, China) and randomly divided into groups as indicated and maintained in specific pathogen free (SPF) condition. Each 1 × 10^6^ of S26 cells containing a specific vector construct were mixed with Matrigel (0.20 v/v, Corning Incorporated, Corning, NY, USA) and subcutaneously injected into dorsal flank of nude mice. Tumor size was measured every week with a caliper, and the tumor volume was determined with the formula: *L* × *W* ^2^ × 0.5, where *L* is the longest diameter and *W* is the shortest diameter. Finally, mice were euthanized, and in vivo solid tumors were dissected for further analysis. All animal experiments were performed under protocols approved by the Institutional Animal Care and Use Committee of Sun Yat‐sen University.

### In Silico Mining of Transcription Factors

TF binding site location was derived from a large collection of ChIP‐seq experiments performed by the ENCODE^[^ ^18^ ^]^ project (https://www.encodeproject.org/) from UCSC database^[^ ^19^ ^]^ (Txn Factor CHIP Track V1, V2, E3, http://genome.ucsc.edu/). All TF sites located between −2500 to +500 bp from the transcription start site (TSS) of RAB26 and ChIP‐seq signal (ChIP score) with over than 50 were retained.

### Statistical Analysis

All data presented as histograms refer to a mean value ± standard deviation (SD) of the total number of independent experiments. Statistical analysis was performed by Student's two‐tailed *t*‐test at a significance level of *p* < 0.05. Survival analysis was performed with SURVIV software.^[^ ^31^ ^]^ Pearson correlation analysis were used to analyze the correlation between the PSI of *GOLIM4* and RBPs expression, and correlation between *E4F1* and *RAB26* expression. Survival curves were calculated by Kaplan–Meier methods, with comparisons performed using the log‐rank test. The key raw data have been deposited in the Research Data Deposit (RDDB2021001596; www.researchdata.org.cn).

## Conflict of Interest

The authors declare no conflict of interest.

## Author Contributions

J.‐X.B conceived the study. C.L. and J.‐X.B designed the experiments and wrote the manuscript. C.L. and X.X. carried out all the experiments. J.C., Y.F., W.P., Y.Z., Y.L., and P.W. assisted with tumor sample preparation. C.L., S.H., and S.L. analyzed the data. H.‐Q.M., B.L, and X.X. provided technical supports.

## Supporting information

Supporting Information

Supplemental Table 1

Supplemental Table 2

Supplemental Table 3

## Data Availability

The data that support the findings of this study are available from the corresponding author upon reasonable request.
