# Supplementary material for: RBFOX2/GOLIM4 Splicing Axis Activates Vesicular Transport Pathway to Promote Nasopharyngeal Carcinogenesis
Source: Adv Sci (Weinh). 2021 Jun 28;8(16):2004852. doi: 10.1002/advs.202004852 (PMC8373120; doi:10.1002/advs.202004852)
Supplement: Supplementary file 1 — Supporting Information [file ADVS-8-2004852-s002.pdf]

© 2021 Wiley-VCH GmbH

ADVANCED SCIENCE
Open Access

Supporting Information

for *Adv. Sci.,* DOI: 10.1002/advs.202004852

RBFOX2/GOLIM4 Splicing Axis Activates Vesicular Transport Pathway to Promote Nasopharyngeal Carcinogenesis

*Chun-Ling Luo, Xiao-Chen Xu, Chu-Jun Liu, Shuai He, Jie-Rong Chen, Yan-Chun Feng, Shu-Qiang Liu, Wan Peng, Ya-Qing Zhou, Yu-Xiang Liu, Pan-Pan Wei, Bo Li, Hai-Qiang Mai, Xiao-Jun Xia, and Jin-Xin Bei**

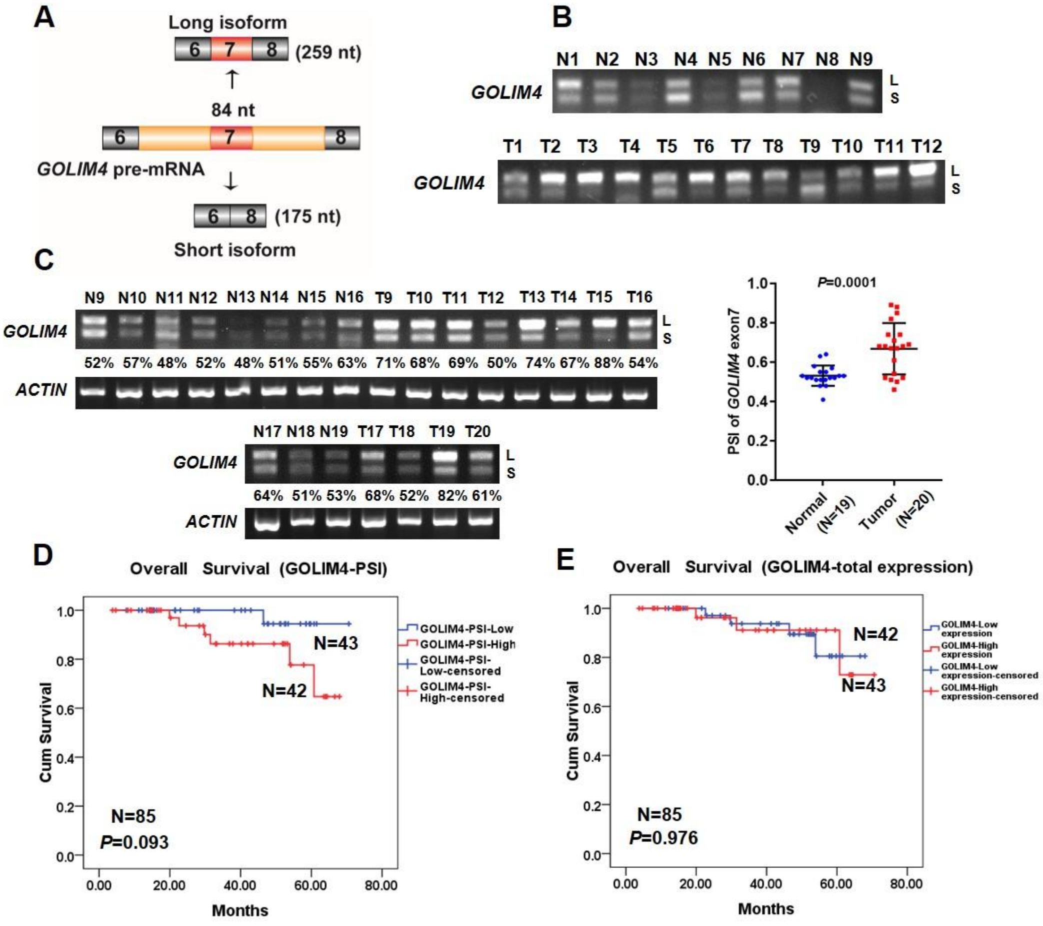

Figure S1.GOLIM4-L is highly transcribed in NPC and associated with poor prognosis. A) Diagram of the splicing variants of *GOLIM4* mRNA and the PCR fragments for the two variants. **B**) RT-PCR showed the splicing pattern of *GOLIM4* exon-7 in NPC tissues (n=85) and control samples (n=10) described in Figure 1A. **C**) RT-PCR showed the transcription level of *GOLIM4* variants in NPC biopsies (T; n=20) and control rhinitis tissues (N; n=19). PSI value of each sample was shown at the bottom. *ACTIN* was used as control.Statistical analysis result was shown at the right. **D**)and **E**) Kaplan-Meier survival curves of overall survival for patients with NPC grouped by PSI level of *GOLIM4-L* (**D**) and the total mRNA expression level of *GOLIM4* (**E**).

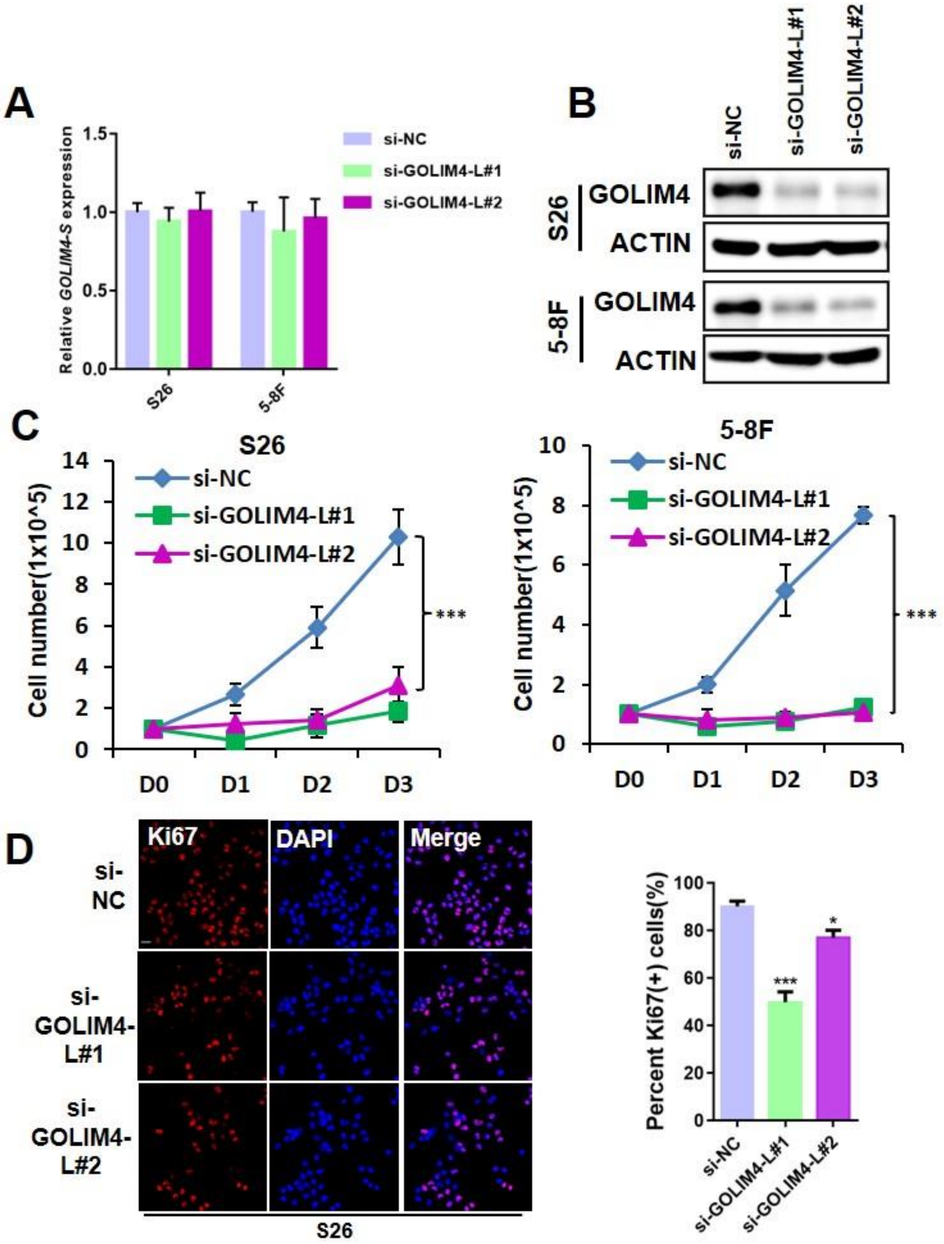

Figure S2.GOLIM4-L deficiency suppressed the proliferation of NPC cells. A)qRT-PCR assays were performed to examine the transcription of *GOLIM4-S* in S26 and 5-8F cells transiently transfected with two siRNAs against *GOLIM4-L* variant. **B**) Western blotting assays showed the protein expression of GOLIM4 in cells described in A. ACTIN was used as control. **C**)Cell proliferation assays were performed using cells described in A. **D**) Fluorescent staining of Ki67 in above S26 cells (left). Corresponding statistical analysis results are presented as mean± SD at the right. Scale bar, 100 μm. **P*<0.05, ***P*<0.01, ****P*<0.001.

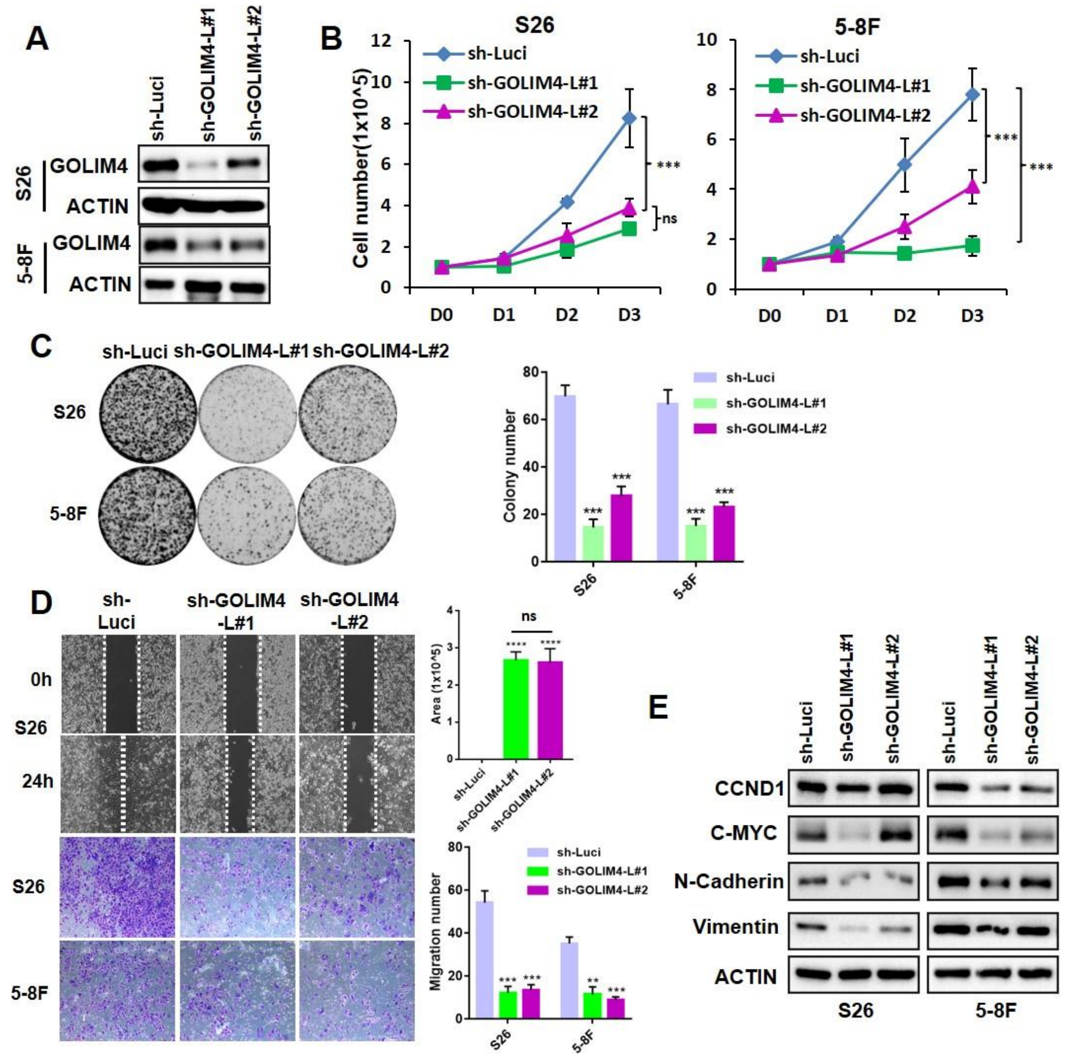

Figure S3.Stable knockdown of GOLIM4-L decreases cell proliferation and migration of NPC cells. A) Western blotting assay showed expression of GOLIM4 in human NPC cell lines S26 and 5-8F cells, which were infected with sh-GOLIM4-L#1, sh-GOLIM4-L#2 or sh-Luci lentivirus. ACTIN was used as control. **B**) Cell proliferation assays were performed using cells described in A. **C**) Colony formation assays were performed with cells described in A. Three independent experiments were performed and representative cells stained with crystal violet were shown (left). The number of focal adhesions was qualified in the bar graph (right). **D**) Wound healing (upper) and transwell assays (bottom) were performed with cells described in A. Statistical analysis results were presented at the right. **E**) Western blotting assay showed the protein levels of CCND1, C-MYC (cell proliferation markers) and N-Cadherin, Vimentin (migration markers). ACTIN was treated as control. Scale bar, 100 μm. *P<0.05, **P<0.01, ***P<0.001, ****P<0.0001.

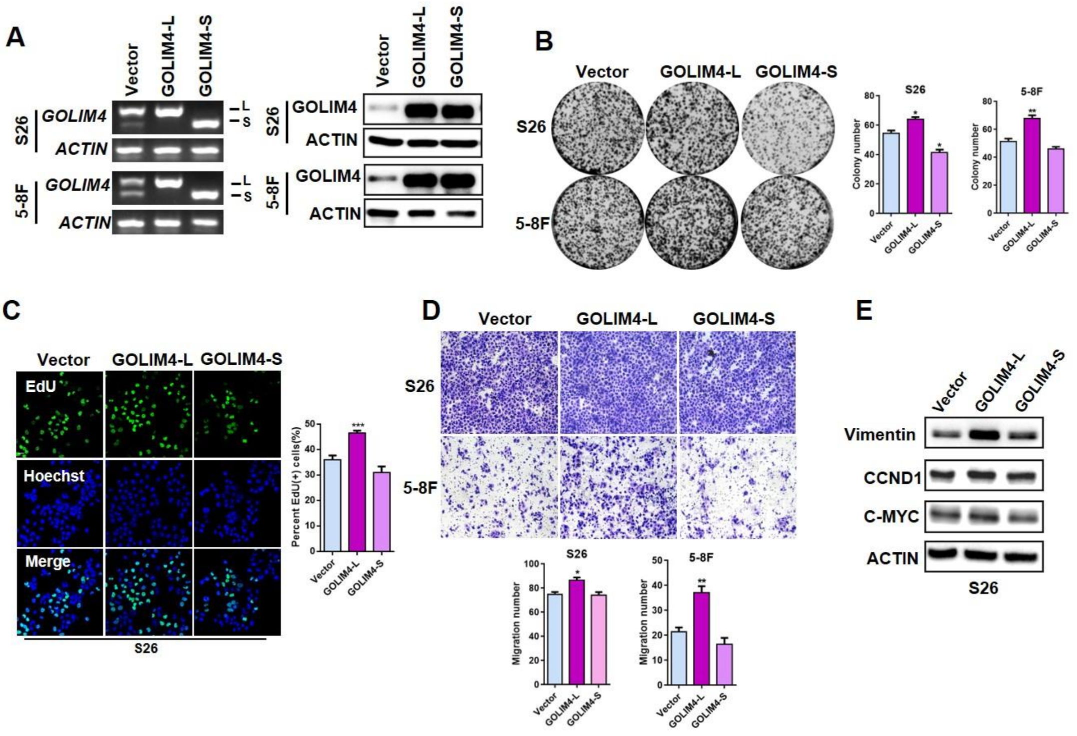

Figure S4.GOLIM4-L promotes cell proliferation and migration of NPC cells. A)RT-PCR (left) and western blotting (right) assays showed mRNA and protein levels of GOLIM4 variants, respectively, in S26 and 5-8F cells. Cells were infected with lentivirus expressing GOLIM4-L/S or empty vector control as indicated. ACTIN was used as control. **B**) Colony formation assays were performed using cells described in A. The number of focal adhesions was qualified in the bar graph (right). **C**) Representative images of EdU staining for S26 cells described in A. Statistical analysis results were presented at the right panel. **D**)Transwell assays were performed with cells described in A. Statistical analysis results were presented at the bottom. **E**) Western blotting assays showed the protein levels of CCND1, C-MYC (cell proliferation markers) and Vimentin (migration marker). ACTIN was treated as control. Scale bar, 100 μm.**P*<0.05, ***P*<0.01, ****P*<0.001.

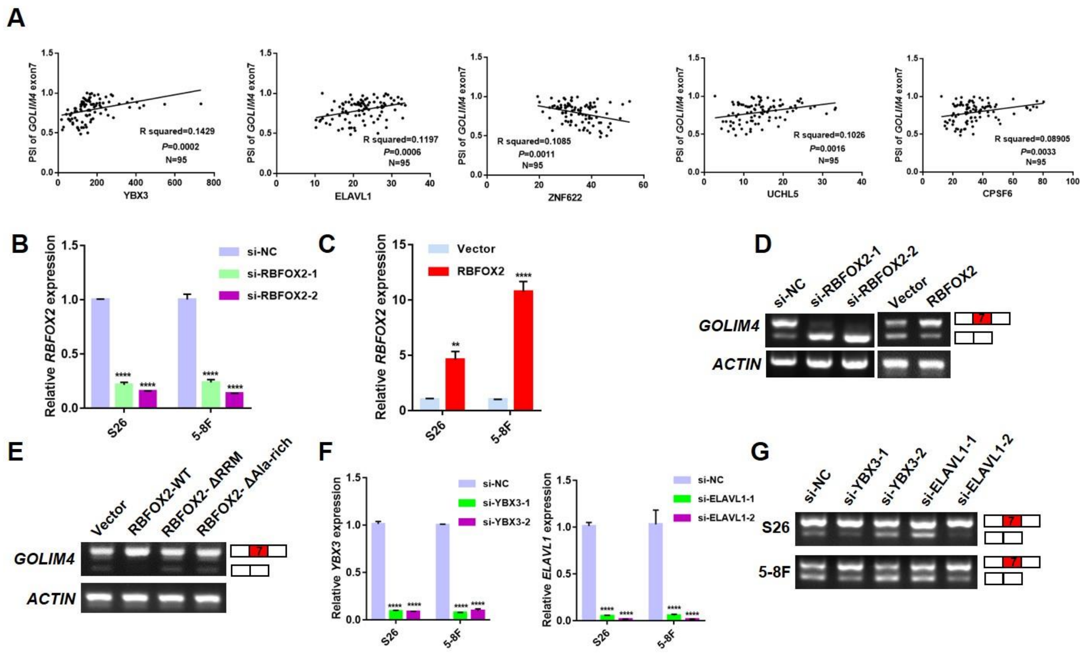

Figure S5.The alternative splicing of *GOLIM4* exon-7 is regulated by splicing factor RBFOX2. A) Correlation between PSI of *GOLIM4-L* and mRNA levels of several candidate RBPs as indicated at the bottom in NPC tissues (n=85) and control samples (n=10). Correlation coefficient R squared and *P* values are based on Pearson's correlation test. **B**) qRT-PCR assays showed the transcription level of *RBFOX2* in human NPC cell lines S26 and 5-8F cells, which were transiently transfected with two siRNAs against RBFOX2. **C**)qRT-PCR assays showed the transcription level *RBFOX2* in human NPC cell lines S26 and 5-8F cells, which were infected with lentivirus expressing RBFOX2 and control vectors. **D**) RT-PCR examination of *GOLIM4* variants in 5-8F cells described above. **E**) RT-PCR assays showed the splicing of *GOLIM4* exon-7 in 293T cells, which were transfected with RBFOX2 and its mutants or control vector. **F**) qRT-PCR assays showed the transcription level of *YBX3* (left) and *ELAVL1* (right) in human NPC cell lines S26 and 5-8F cells, which were transiently transfected with YBX3 and ELAVL1 siRNAs and control siRNA. **G**) RT-PCR was performed to detect the splicing pattern of *GOLIM4* exon-7 in cells described in F. **P*<0.05, ***P*<0.01, ****P*<0.001, *****P*<0.0001.

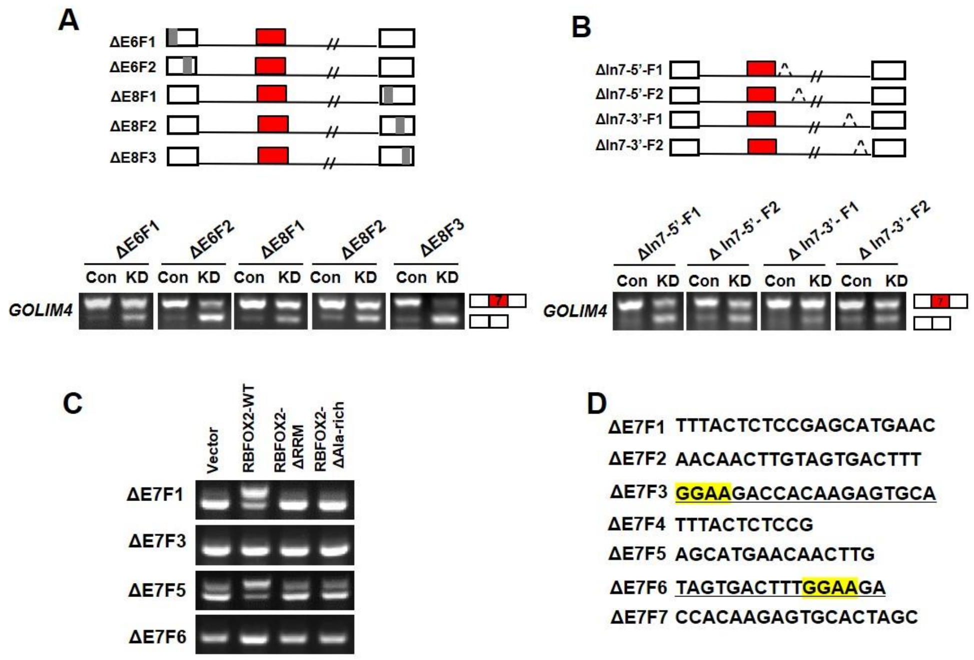

Figure S6.Examination of binding motif of RBFOX2 in the exon-7 to promote the inclusion of *GOLIM4* exon-7.A) Several fragments of *GOLIM4* minigene with indicated deletions (grayed) on exon-6 and exon-8 were generated (top). RT-PCR examined the transcription of *GOLIM4* variants in 293T cells, which were co-transfected GOLIM4 mutant plasmids and si-RBFOX2 (KD) or si-NC (Con, as Control). **B**) Several fragments of *GOLIM4* minigene with indicated deletions (dashed triangle) on intron-7 were generated (top). RT-PCR examined the transcription of *GOLIM4* variants in 293T cells, which were co-transfected with si-RBFOX2 or si-NC along with mutant plasmids indicated on top. **C**)RT-PCR was performed to examine the splicing pattern of *GOLIM4* in 293T cells, which were co-transfected with GOLIM4 mutant plasmids and wild-type, mutant RBFOX2, or control vector. **D**) Sequences of each fragment were presented. The GGAA sequence was shaded in yellow.

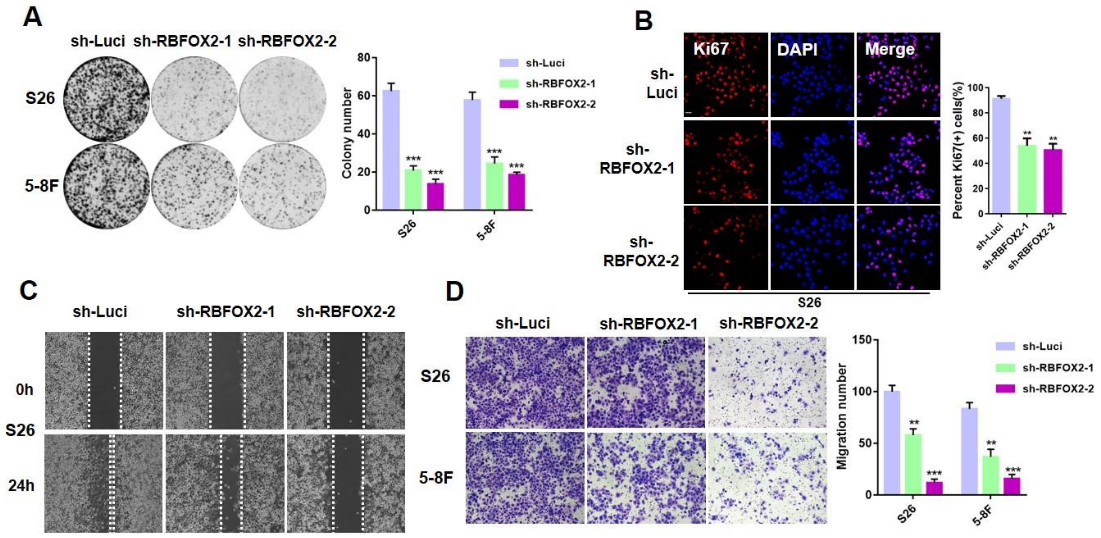

Figure S7. RBFOX2 acts as a tumorigenesis regulator in NPC cells. A)Colony formation assays were performed with S26 and 5-8F cellsinfected with sh-RBFOX2-1, sh-RBFOX2-2 or sh-Luci lentivirus. Quantification of colony numbers was shown at the right. Three independent experiments were performed and error bars indicate SD of mean. **B**)Fluorescent staining of Ki67 assays with S26 cellsdescribed in A. Corresponding statistical analysis results were presented at the right. **C**)and **D**) Wound healing (C) and transwell assays (D) were performed with cells described in A. Statistical analysis results were shown on the right. Scale bar, 100 μm. *P<0.05, **P<0.01, ***P<0.001.

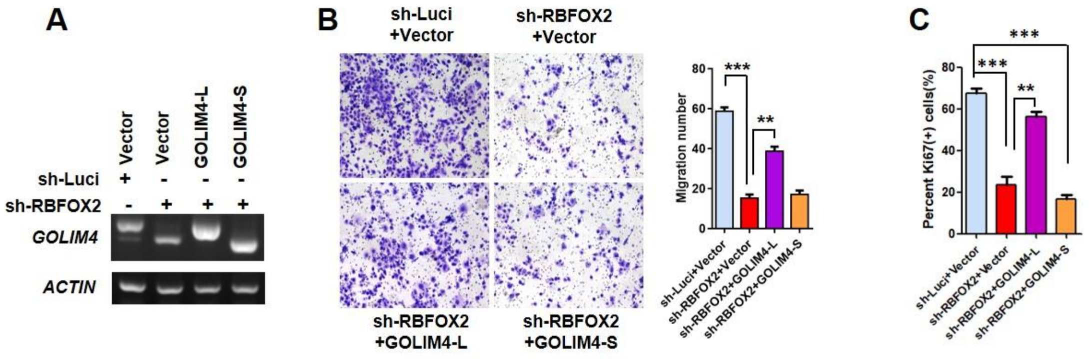

Figure S8.Overexpression of GOLIM4-L partly rescues the inhibition on the migration and cell proliferation of RBFOX2-knockdown cells. A) Expression of *GOLIM4* variants was determined using RT-PCR. S26 cells expressing shRBFOX2 or sh-Luci (control)were infected with lentivirus expressing GOLIM4-L, GOLIM4-S or empty vectors. **B**)Transwell assayswere performed with cells described A. Statistical analysis results were shown at the right. **C**) Statistical analysis results with Ki-67 staining described in Figure4H were shown. Scale bar, 100 μm.*P<0.05, **P<0.01, ***P<0.001.

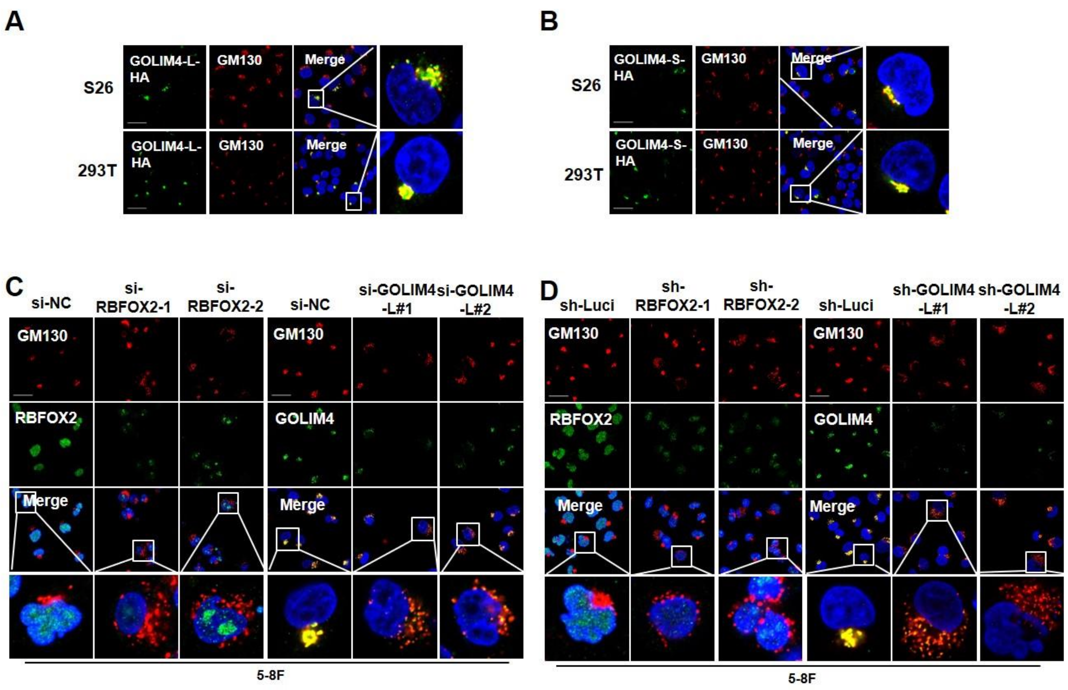

Figure S9.RBFOX2 or GOLIM4-L deficiency leads to fragmentation of Golgi apparatus in NPC cells.A)and**B)** Immunofluorescent staining with indicated antibodies showed the localization of GOLIM4-L (**A,** green) and GOLIM4-S (**B,** green) with GM130 (red) in S26 and 293T cells transfected with GOLIM4-L-HA or GOLIM4-S-HA plasmids, respectively. **C)**Immunofluorescent staining of endogenous GM130 (red) together with RBFOX2 (green) or GOLIM4 (green) in 5-8F cells transfected with RBFOX2, GOLIM4-L siRNAs or control siRNAs indicated on top.**D)** Immunofluorescent staining of endogenous GM130 (red) together with RBFOX2 (green) or GOLIM4 (green) in 5-8F cells infected with lentivirus expressing RBFOX2, GOLIM4-L or control shRNAs indicated on top.Scale bar, 100 μm.

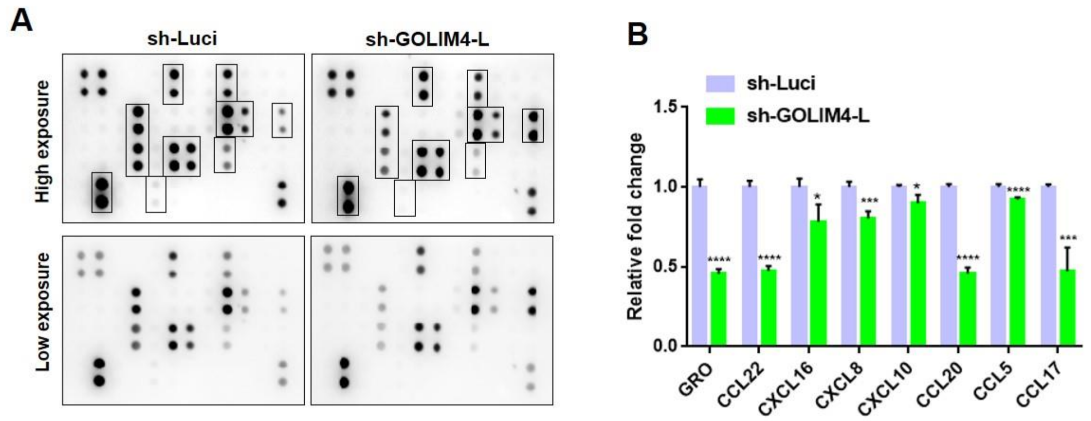

Figure S10. Knockdown of GOLIM4-L reduces the secretion of cancer growth-promoting factors. Human chemokine arrays were performed to measure the expression of 38 human chemokines in S26 cells expressing GOLIM4-L or control shRNAs. **A**) Representative images of the array membranes are shown. **B**) The quantitation was presented at the right. **P*<0.05, ***P*<0.01, ****P*<0.001, *****P*<0.0001.

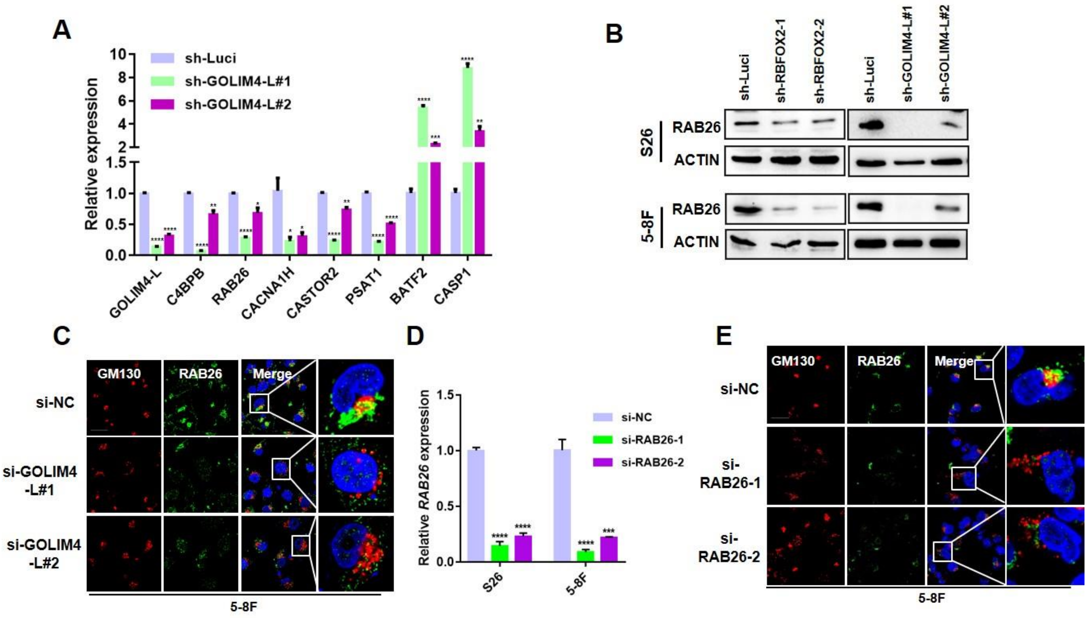

Figure S11.GOLIM4-L deficiency decreases the expression and Golgi localization of RAB26 in NPC cells.A) qRT-PCR validation of eight candidate genes derived from the RNA-seq analysis in S26 cells infected with GOLIM4-L shRNAs. The experiments were repeatedat least three times, and results are shown as mean± SD.**B**)Western blot assay showed the protein level of RAB26 in S26 and 5-8F cells infected with RBFOX2 or GOLIM4 shRNAs. **C**) Immunofluorescent staining of endogenous GM130 (red) and RAB26 (green) in 5-8F cells transfected with GOLIM4-L siRNAs. **D**)qRT-PCRanalysis showed the expression of RAB26 in S26 and 5-8F cells, which were transfected with RAB26 siRNAs or control siRNA. **E**) Immunofluorescent staining of endogenous GM130 (red) and RAB26 (green) in 5-8F cells described in D.Scale bar, 100 μm. **P*<0.05, ***P*<0.01, ****P*<0.001, *****P*<0.0001.

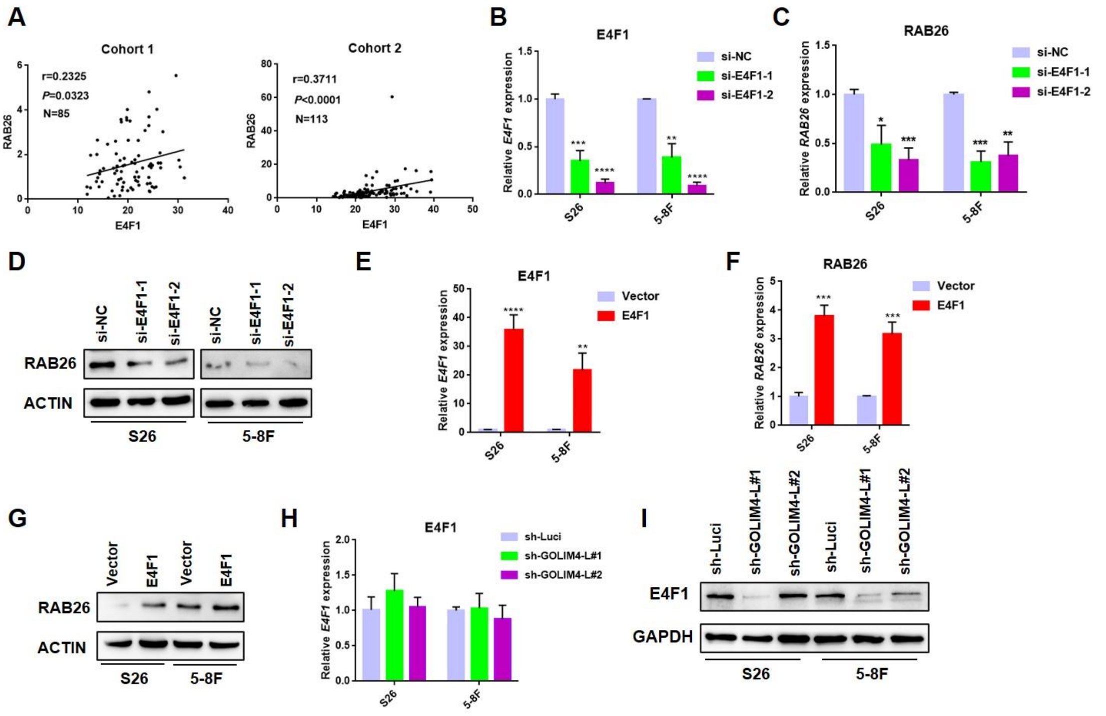

Figure S12. GOLIM4-L may regulate the transcriptional level of *RAB26* through atranscription factor E4F1. **A**) Correlation between the expression of RAB26 and E4F1 in transcription data withtwo NPC cohorts. **B**)and **C**) qRT-PCR assays showed the mRNA expression of *E4F1* (B) and *RAB26* (C) in S26 and 5-8F cells, which were transiently transfected with two siRNAs against E4F1. **D)** Western blotting assays showed the protein level of RAB26 in S26 and 5-8F cells described in B. ACTIN was used as control. **E**)and **F**) qRT-PCR assays showed the mRNA expression of *E4F1* (E) and *RAB26* (F) in S26 and 5-8F cells infected with lentivirus expressing E4F1. **G**) Western blotting assays showed the protein level of RAB26 in S26 and 5-8F cells described in E. ACTIN was used as control. **H**)and **I**) qRT-PCR and western blotting assays showed the mRNA (H) and protein expression (I) of E4F1 in S26 and 5-8F cells infected with lentivirus expressing GOLIM4-L shRNAs or control shRNA. GAPDH was treated as control. *P<0.05, **P<0.01, ***P<0.001, ****P<0.0001.

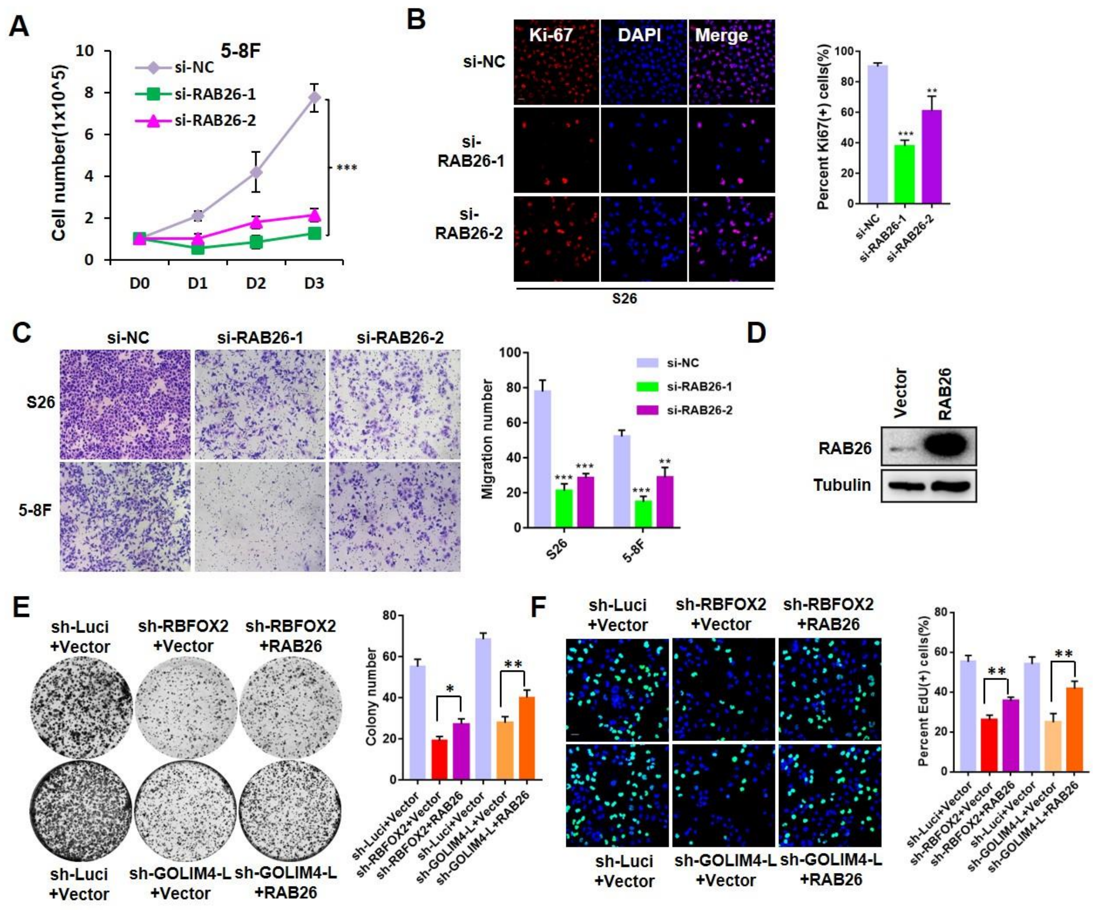

Figure S13.RAB26 is required for cell proliferation and migration of NPC cells.A) Cell proliferation assays were performed using 5-8F cells,which were transfected with RAB26 siRNAs or control siRNA.**B**) Representative images of Ki-67 staining assays (left) in S26 cellstransfected with RAB26 siRNAs or control siRNA.**C**) Transwell assays were performed with cells described in A and B. Statistical analysis results were shown at the right. **D**) Western blotting assay showed the protein levels of RAB26 in S26 cells infected with lentivirus expressing RAB26 or control vectors. Tubulin was treated as control. **E**)Colony formation assayswere performed with S26 cells expressing sh-RBFOX2, sh-GOLIM4-L or sh-Luci (control)were infected with lentivirus expressing RAB26 or empty vectors. Quantification of colony numbers was shown at the right. Three independent experiments were performed and error bars indicate SD of mean.**F**) Representative images of EdU staining assays (left) for cells described in E and corresponding statistics are shown at the right. Scale bar, 100 μm. **P*<0.05, ***P*<0.01, ****P*<0.001, *****P*<0.0001.

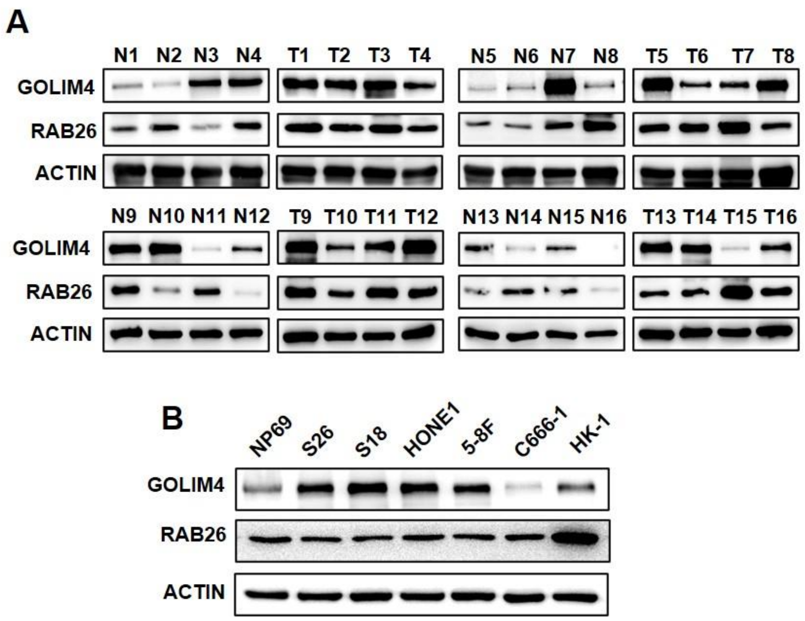

Figure S14. Western blotting assays reveal protein expression of GOLIM4 and RAB26 in NPC tissues (A) and cell lines (B). Antibodies used are indicated at the left. NPC (T; n=16), control samples (N; n=16), or cell line identities are indicated on the top. NP69, transformed nasopharyngeal epithelium cells; ACTIN, control protein.
