# Supplementary material for: RBFOX2/GOLIM4 Splicing Axis Activates Vesicular Transport Pathway to Promote Nasopharyngeal Carcinogenesis
Source: Adv Sci (Weinh). 2021 Jun 28;8(16):2004852. doi: 10.1002/advs.202004852 (PMC8373120; doi:10.1002/advs.202004852)
Supplement: Supplementary file 4 — Supplemental Table 3 [file ADVS-8-2004852-s001.docx]

**Table S4. Primer-id sequence (5'-3')**

| GOLIM4-E6-F | AAGGAAAGCACACCAAGACA |
| --- | --- |
| GOLIM4-E8-R | TGCTGTGTTGTAACCTTGTGG |
| GOLIM4-L-F | CTCTCCGAGCATGAACAACTT |
| GOLIM4-L-R | TGGGTCACATTTTGCTGTTC |
| GOLIM4-S-F | TCAAGATGTCAAGACTCAAGTTGC |
| GOLIM4-S-R | TATTTCGAGACACCTCCTGC |
| GOLIM4-EcoR1-F | CCGGAATTCGACTATGGGAAACGGGATGTG |
| GOLIM4-BamH1-R | CGCGGATCCCTACATTTCAGCTCTTCGATGTG |
| GOLIM4-BamH1-F | CGCGGATCCGACTATGGGAAACGGGATGTG |
| GOLIM4-EcoR1-R | CCGGAATTCTGCCGCTACATTTCAGCTCTTC |
| RBFOX2-F | \| AAGCCCAGTAGTTGGAGCTG \| \| --- \| |
| RBFOX2-R | GGGAAGCCAGGAACTAAAGG |
| RBFOX2-BamH1-F | CGCGGATCCATGGCGGAGGGCGCCCAGCCGCAT |
| RBFOX2-Xho1-R | CCGCTCGAGTCAGTAGGGGGCAAATCGG |
| RBFOX2-D1-F | AGAGTAAATCTACCCCGCGTGTAATGACCAATAAG |
| RBFOX2-D1-R | CTTATTGGTCATTACACGCGGGGTAGATTTACTCT |
| RBFOX2-D2-F | TGGCTTCCCTTACCCTACTAGTTTATACCGAGGTGGCTA |
| RBFOX2-D2-R | TAGCCACCTCGGTATAAACTAGTAGGGTAAGGGAAGCCA |
| RAB26-BamH -F | CCGGGATCCATGTCCAGGAAGAAGACCCCCAA |
| RAB26-EcoR1-R | CGCGAATTCGGACTGAGCTCAGCCAGGTT |
| RAB26- EcoR1 -F | CCGGAATTCATGTCCAGGAAGAAGACCCCCAA |
| RAB26- BamH1-R | CGCGGATCCGGACTGAGCTCAGCCAGGTT |
| E4F1-EcoR1-F | CCGGAATTCATGGAGGGCGCGATGGCAGT |
| E4F1-BamH1-R | CGCGGATCCATGCTAGACGATGACCGTCTG |
| RAB26-F | CGATTCAAGGATGGTGCTTT |
| RAB26-R | CCGGTAGTAGGCATGGGTAA |
| CACNA1H-F | TACTCGTTGGACGGACACAA |
| CACNA1H-R | AAGCACAGCAGAAGGACGTT |
| C4BPB-F | TAGGGACTGTGACCCTCCTG |
| C4BPB-R | TGGATCAACTTGCAGACTGG |
| BATF2-F | GCAGGGGTCTTCCTCTAAGC |
| BATF2-R | GCTGCTGAGAGAGCAGGTTT |
| PSAT1-F | AGAATCTTGTGCGGGAATTG |
| PSAT1-R | CCCAAGTTTAGGGTGAACGA |
| CASTOR2-F | TTTGATGAGTGTGGCATCGT |
| CASTOR2-R | TTCTCTGCTTGGCTGACCTT |
| CASP1-F | GGAAACAAAAGTCGGCAGAG |
| CASP1-R | ACGCTGTACCCCAGATTTTG |
| E4F1-F | CAAGTGCTCCAAGTGTGGAA |
| E4F1-R | CTATAGGCTCGCCTGTCACC |
| GOLIM4-RIP-E6-F | GACAACTAAGGAAAGCACACCA |
| GOLIM4-RIP-E6-R | CCAAATAAAGCAGGCAAACA |
| GOLIM4-RIP-IN6-F | TGATATTTGGGTAGCACGTTTT |
| GOLIM4-RIP-IN6-R | TCTTCTTTGGCTGATCGTGA |
| GOLIM4-RIP-E7-F | CTCTCCGAGCATGAACAACTT |
| GOLIM4-RIP-E7-F | AGAACCCACAGAGGCTGCTA |
| GOLIM4-RIP-IN7-5’-F | GTAGCAGCCTCTGTGGGTTC |
| GOLIM4-RIP-IN7-5’-R | AAAGCATGCATGATCCTCAG |
| GOLIM4-RIP-IN7-3’-F | GCATGCGCTCAAAAATGAT |
| GOLIM4-RIP-IN7-3’-R | GATGGGCATTTGGGTACACT |
| GOLIM4-RIP-E8-F | AGCCTTCGAAAACCTGATCC |
| GOLIM4-RIP-E8-F | ACCTCTCGGGTTGGCTTCT |
| GOLIM4-minigene-Kpn1-F1-F | CGGGGTACCAGAGACTGTATACAATTTGAGAG |
| GOLIM4-minigene-BamH1-F1-R | CGCGGATCCGAGGGTGTGTAGGCTGGAAT |
| GOLIM4-minigene-BamH1-F2-F | CGCGGATCCCATTCTAGCCAATTAGGGATTC |
| GOLIM4-minigene-Xho1-F2-R | CCGCTCGAGTTACCTCCTGCACCTCTCG |
| GOLIM4-minigene-F | GGTACCAGAGACTGTATACA |
| GOLIM4-minigene-R | CTCGAGTTACCTCCTGCACCT |
| GOLIM4-E6-dele1-F | GGTACCAGAGACTGTATAGCACACCAAGACATAC |
| GOLIM4-E6-dele1-R | GTATGTCTTGGTGTGCTATACAGTCTCTGGTACC |
| GOLIM4-E6-dele2-F | AGACAACTAAGGAAAAGATGTCAAGgtaa |
| GOLIM4-E6-dele2-R | ttacCTTGACATCTTTTCCTTAGTTGTCT |
| GOLIM4-E8-dele1-F | tgcagACTCAAGTTGTCGAAAACCTGATCCA |
| GOLIM4-E8-dele1-R | TGGATCAGGTTTTCGACAACTTGAGTctgca |
| GOLIM4-E8-dele2-F | TAGGATTCCAAGCCTCATTCTCCACAAGGT |
| GOLIM4-E8-dele2-R | ACCTTGTGGAGAATGAGGCTTGGAATCCTA |
| GOLIM4-E8-dele3-F | TGTGACCCAGGTGGCAGGTGCAGGAGgtaa |
| GOLIM4-E8-dele3-R | ttacCTCCTGCACCTGCCACCTGGGTCACA |
| GOL-IN7-5’-dele1-F | gcagcctctgtgggttcttcgtccctacatgcagt |
| GOL-IN7-5’-dele1-R | actgcatgtagggacgaagaacccacagaggctgc |
| GOL-IN7-5’-dele2-F | gatcatttctttccactcttccagcctacacaccc |
| GOL-IN7-5’-dele2-R | gggtgtgtaggctggaagagtggaaagaaatgatc |
| GOL-IN7-3’-dele1-F | catcacagaacggtcgattaagcatgcccaa |
| GOL-IN7-3’-dele1-R | ttgggcatgcttaatcgaccgttctgtgatg |
| GOL-IN7-3’-dele2-F | tttgcatgcgctcaatatgaatgggcattca |
| GOL-IN7-3’-dele2-R | tgaatgcccattcatattgagcgcatgcaaa |
| GOLIM4-E7-1-F | CAACAGCATAAGAATAACTTGTAGTGACTT |
| GOLIM4-E7-1-R | AAGTCACTACAAGTTATTCTTATGCTGTTG |
| GOLIM4-E7-2-F | TCTCCGAGCATGAACGGAAGACCACAAGAGT |
| GOLIM4-E7-2-R | ACTCTTGTGGTCTTCCGTTCATGCTCGGAGA |
| GOLIM4-E7-3-F | ACTTGTAGTGACTTTCTAGCTGCTGCACAG |
| GOLIM4-E7-3-R | CTGTGCAGCAGCTAGAAAGTCACTACAAGT |
| GOLIM4-E7-4-F | agCAACAGCATAAGAAGCATGAACAACTTG |
| GOLIM4-E7-4-R | CAAGTTGTTCATGCTTCTTATGCTGTTGct |
| GOLIM4-E7-5-F | AGAATTTACTCTCCGTAGTGACTTTGGAAGA |
| GOLIM4-E7-5-R | TCTTCCAAAGTCACTACGGAGAGTAAATTCT |
| GOLIM4-E7-6-F | AGCATGAACAACTTGCACAAGAGTGCACTAG |
| GOLIM4-E7-6-R | CTAGTGCACTCTTGTGCAAGTTGTTCATGCT |
| GOLIM4-E7-7-F | TGACTTTGGAAGACAGCTGCTGCACAGgt |
| GOLIM4-E7-7-R | acCTGTGCAGCAGCTGTCTTCCAAAGTCA |
| GOLIM4-E7-3-IN-F | ACTTGTAGTGACTTTGGAAGGAACTAGCTGCTGCACAG |
| GOLIM4-E7-3-IN-R | CTGTGCAGCAGCTAGTTCCTTCCAAAGTCACTACAAGT |
| GOLIM4-E7-3-INR-F | ACTTGTAGTGACTTTctgatctgCTAGCTGCTGCACAG |
| GOLIM4-E7-3-INR-R | CTGTGCAGCAGCTAGcagatcagAAAGTCACTACAAGT |
| si-NC (5’-3’) | UUCUCCGAACGUGUCACGUTT |
| si-GOLIM4-L#1(5’-3’) | CUCCGAGCAUGAACAACUUTT |
| si-GOLIM4-L#2 (5’-3’) | AUGUCAAGCAACAGCAUAATT |
| si-RBFOX2-1 (5’-3’) | CUGACACUCUACGGAAGUATT |
| si-RBFOX2-2 (5’-3’) | GGCAAAAUCCUAGAUGUAGTT |
| si-YBX3-1 (5’-3’) | UCCUAACGCUCCUUCACAATT |
| si-YBX3-2 (5’-3’) | ACGGAAAUAUCUGCGCAGUTT |
| si-ELAVL1-1 (5’-3’) | GAGUUACGAAGCCUGUUCATT |
| si-ELAVL1-2 (5’-3’) | GGAUCAGACUACAGGUUUGTT |
| si-RAB26-1 (5’-3’) | GUGUUACCCAUGCCUACUATT |
| si-RAB26-2 (5’-3’) | CAGCCAUAGCAAAGGAGUUTT |
| si-E4F1-1 (5’-3’) | CACGCAUCTGACCUUGUUGTT |
| si-E4F1-2 (5’-3’) | GAACAAGGAUGGCCGCUAU TT |
| sh-Luci-F | CCGGTTCCTGGAACAATTGCTTTTACTCGAGTAAAAGCAATTGTTCCAGGAATTTTTG |
| sh-Luci-R | AATTCAAAAATTCCTGGAACAATTGCTTTTACTCGAGTAAAAGCAATTGTTCCAGGAA |
| sh-GOLIM4-L#1-F | CCGGAACTCCGAGCATGAACAACTTCTCGAGAAGTTGTTCATGCTCGGAGTTTTTTTG |
| sh-GOLIM4-L#1-R | AATTCAAAAAAACTCCGAGCATGAACAACTTCTCGAGAAGTTGTTCATGCTCGGAGTT |
| sh-GOLIM4-L#2-F | CCGGAAATGTCAAGCAACAGCATAACTCGAGTTATGCTGTTGCTTGACATTTTTTTTG |
| sh-GOLIM4-L#2-R | AATTCAAAAAAAATGTCAAGCAACAGCATAACTCGAGTTATGCTGTTGCTTGACATTT |
| sh-RBFOX2-1-F | CCGGCGGGTTCGTAACTTTCGAGAACTCGAGTTCTCGAAAGTTACGAACCCGTTTTTG |
| sh-RBFOX2-1-R | AATTCAAAAACGGGTTCGTAACTTTCGAGAACTCGAGTTCTCGAAAGTTACGAACCCG |
| sh-RBFOX2-2-F | CCGGTTGGCGCTGTGGCGAGTTTATCTCGAGATAAACTCGCCACAGCGCCAATTTTTG |
| sh-RBFOX2-2-R | AATTCAAAAATTGGCGCTGTGGCGAGTTTATCTCGAGATAAACTCGCCACAGCGCCAA |

**Table S4. List of specific primers.**
